# Qijia Rougan Formula alleviates liver fibrosis by inhibiting NLRP3-mediated pyroptosis and regulating macrophage polarization

**DOI:** 10.3389/fimmu.2026.1796042

**Published:** 2026-05-05

**Authors:** Xin Ding, Baixue Li, Hongfei Song, Chenhao Liu, Zhenglong Zheng, Jibin Liu, Li Wen, Quansheng Feng

**Affiliations:** 1 College of Basic Medical Sciences, Chengdu University of Traditional Chinese Medicine, Chengdu, China; 2 College of Traditional Chinese Medicine, Inner Mongolia Medical University, Hohhot, China

**Keywords:** macrophage polarization, NLRP3 inflammasome, pyroptosis, Qijia Rougan Formula, stage F3 liver fibrosis

## Abstract

**Background:**

Stage F3 liver fibrosis represents a critical phase for intercepting the progression toward irreversible cirrhosis. Despite its clinical significance, effective interventions remain scarce. Clinical observations indicate that the traditional Chinese medicine (TCM) formula Qijia Rougan Formula (QRF) exhibits pronounced anti-fibrotic efficacy specifically at Stage F3. Preliminary data suggest that QRF may exert immunomodulatory and anti-fibrotic effects by targeting NLRP3 inflammasome/GSDMD axis. Its precise mechanistic underpinnings warrant further elucidation.

**Methods:**

C57BL/6 mice were administered CCl_4_ to establish stage F3 model. Following a 6-week therapeutic regimen, hepatic structural integrity and functional recovery were assessed. A multi-tiered investigative strategy was employed: molecular docking predicted ligand-protein interactions, while transcriptomic profiling identified differentially expressed genes (DEGs). *In vitro*, LPS/ATP-primed macrophages were treated with QRF-medicated serum. The ultrastructural hallmarks of pyroptosis were visualized using transmission electron microscopy (TEM). A functional rescue experiment was performed using NLRP3-specific agonist Nigericin (NIG) to establish causality.

**Results:**

High-dose QRF significantly ameliorated hepatic dysfunction and histopathological injury in F3 model mice. Integrated in molecular docking and transcriptomic analysis identified the NLRP3-mediated pyroptotic pathway as QRF’s primary therapeutic target. Specifically, QRF suppressed NLRP3, Caspase-1, and GSDMD expression, thereby curtailing pro-inflammatory cytokines (IL-1β, IL-18) and LDH release. Concurrently, QRF facilitated a macrophage phenotypic switch from the pro-inflammatory M1 to the anti-inflammatory M2 phenotype. TEM confirmed that QRF preserved membrane integrity and mitigated organelle damage. Notably, the anti-pyroptotic efficacy of QRF was abolished by NIG, confirming its dependence on NLRP3 inhibition.

**Conclusions:**

QRF exerts significant anti-fibrotic activity in stage F3 fibrosis by inhibiting NLRP3/Caspase-1/GSDMD mediated macrophage pyroptosis. By quenching this pathway, QRF curtails the inflammatory cascade and promotes M1-to-M2 macrophage polarization, thereby optimizing intrahepatic immune microenvironment and retarding fibrotic progression. Utilizing the reliable established F3 animal model, these findings systematically elucidate the QRF’s key targets and provide experimental foundation for developing stage-specific therapeutic strategies.

## 1 Introduction

Liver fibrosis, a hallmark of chronic liver disease progression, is characterized by excessive extracellular matrix (ECM) deposition resulting from a dysregulated wound-healing response, now recognized as a chronic, inflammation-driven, immune-mediated process (1–3). If left untreated, it may progress to cirrhosis and liver failure, typically over 10–20 years (4). According to internationally accepted METAVIR histologic scoring system (5, 6), which assesses fibrosis severity based on liver histology, fibrosis is staged from F0 to F4. Accumulating clinical evidence indicates that stage-specific interventions are more effective in attenuating fibrosis (7–10), highlighting the therapeutic relevance of targeting hepatic stellate cells (HSCs) and associated inflammatory and immune pathways. Stage F3, categorized as advanced fibrosis, is characterized by extensive bridging fibrosis without cirrhotic nodules, reflects substantial ECM accumulation and architectural distortion preceding pseudolobule formation. Timely intervention at this stage may improve prognosis (4). However, without appropriate treatment, progression to irreversible cirrhosis remains likely. Therefore, stage F3 represents a critical transitional stage with substantial potential for fibrosis amelioration, underscoring the need for in-depth investigation and the development of targeted therapeutic strategies.

Currently, no biologic or chemical agents with definitive efficacy are available for the clinical treatment of liver fibrosis. Traditional Chinese medicine (TCM), characterized by its multi-target properties and relatively low toxicity, has shown considerable antifibrotic potential. TCM exert effects by regulating cellular functions, attenuating oxidative stress and inflammation, and maintaining ECM homeostasis (11–15). Emerging evidence further highlights their immunomodulatory roles, particularly in shaping the hepatic immune microenvironment through macrophage polarization (2, 3). Clinically, guideline-recommended formulations such as Fuzheng Huayu Formula and Compound Biejia Rougan Tablets have demonstrated efficacy in delaying liver fibrosis progression (6, 15), with increasing evidence indicating stage-dependent efficacy, especially in stage F3/F4 (16, 17). Qijia Rougan Formula (QRF; Patent No. ZL 2023 1 1665187.2), developed by our research group, has demonstrated promising antifibrotic effects. Initial clinical observations (18) and the subsequent experimental studies (19, 20) have shown that QRF can alleviate hepatic injury and collagen deposition while inhibit HSCs activation. Furthermore, our previous randomized controlled trial (RCT) demonstrated that QRF significantly improved fibrosis severity and clinical symptoms, with particularly pronounced efficacy in stage F3 fibrosis (21). Despite these findings, the mechanisms underlying the stage-specific efficacy of QRF, particularly at the critical F3 stage, remain poorly understood.

This study focuses on stage F3 liver fibrosis and aims to elucidate the molecular mechanisms underlying the pronounced antifibrotic effects of QRF at this critical stage. Previous clinical and experimental studies have demonstrated that QRF significantly reduces the expression of key pyroptosis executors, including gasdermin D (GSDMD) and multiple caspases, while inhibiting the CXCL12/CXCR4/TXNIP/NLRP3 signaling axis (21–23). These findings suggest that its antifibrotic effects may be associated with suppression of NLRP3 inflammasome-mediated pyroptosis. Pyroptosis is a form of inflammasome-mediated programmed cell death, primarily driven by the canonical NLRP3/Caspase-1/GSDMD pathway, which induces membrane pore formation and promotes the maturation and release of pro-inflammatory cytokines such as IL-1β and IL-18. This process contributes to HSCs activation and ECM deposition, thereby facilitating fibrosis progression (24, 25). In addition, macrophage pyroptosis has been recognized as an important driver of hepatic inflammation and fibrosis (26, 27). Furthermore, macrophage polarization is a critical regulator of the hepatic immune microenvironment. Pro-inflammatory M1 and reparative M2 phenotypes exert distinct effects on HSCs activation and tissue remodeling, and modulation of their polarization (inhibition of M1 or promotion of M2) has been shown to attenuate liver fibrosis (2, 3). However, how QRF alleviates liver fibrosis *via* coordinated modulation of macrophage pyroptosis and polarization, particularly at the advanced F3 stage, remain inadequately investigated.

To address this, molecular docking was first employed to predict interactions between QRF-derived compounds and key proteins involved in pyroptosis. A CCl_4_-induced mouse model mimicking F3-stage fibrosis was then established to evaluate the therapeutic effects of QRF. Transcriptomic analysis of liver tissues was further conducted to assess the regulation of the NLRP3/Caspase-1/GSDMD pathway and characterize the expression profiles of pyroptosis-related genes. Concurrently, the effects of QRF on macrophage polarization in fibrotic liver were investigated. This study seeks to systematically decipher a previously unexplored immunomodulatory mechanism through which QRF exhibits antifibrotic effects at F3 stage—namely by preventing the activation of the canonical pyroptosis pathway mediated by NLRP3, which consequently reduces macrophage pyroptosis and modulates macrophage polarization toward an anti-fibrotic phenotype, thereby mitigating inflammation and activation of HSCs. These findings are expected to systematically elucidate the key targets of QRF and provide a compelling experimental foundation for developing stage-specific therapeutic strategies.

## 2 Materials and methods

### 2.1 QRF preparation

QRF was formulated from eight medicinal herbs: *Astragalus membranaceus* (Fisch.) Bunge (Chinese name: Huangqi, HQ), *Angelica sinensis* (Oliv.) Diels (Chinese name: Danggui, DG), *Curcuma phaeocaulis* Valeton (Chinese name: Ezhu, EZ), *Trionycis Carapax* (Chinese name: Biejia, BJ), *Carthamus tinctorius* L. (Chinese name: Honghua, HH), *Sparganium stoloniferum* Buch.-Ham. (Chinese name: Sanleng, SL), *Prunus davidiana* (Carr.) Franch. (Chinese name: Taoren, TR), and *Glycyrrhiza uralensis* Fisch. (Chinese name: Gancao, GC). Detailed botanical identification and quality control have been reported previously (21, 28). All crude materials were supplied by Inner Mongolia Ruitai Pharmaceutical Co., Ltd. (Hohhot, China) and authenticated by the Department of Pharmacognosy at Inner Mongolia Medical University. The herbs were combined at a ratio of HQ: DG : EZ: BJ : HH: SL : TR: GC = 30:9:15:15:18:15:15:5. BJ was pre-soaked in purified water for 30 min, followed by immersion of the full mixture in 10-fold water for 1 h and three rounds of reflux extraction (1 h each). The extracts were pooled, concentrated under reduced pressure, and freeze-dried to yield a final extraction rate of 20.12%. Silymarin (Yuanye: SS8540-20mg) served as the positive control. Animal doses were calculated based on body surface area conversion (29).

### 2.2 Preparation of drug-containing serum

Drug-containing serum was prepared using SD rats (30, 31). Seventeen healthy male SPF-grade rats were acclimatized for one week under standard conditions (as described in Section 2.1) and then randomly divided into a blank control group (n=5) and a treatment group (n=12). Dosing was calculated based on body surface area-equivalent doses and administered via gavage at 5–10 times the calculated dose to ensure adequate concentrations of the active drug component in serum. The treatment group received gavage twice daily (5 h intervals) for one week. The rats were fasted for 12 h prior to the final dose (with free access to water), and blood was collected via the abdominal aorta 1.5–3 h after dosing. Whole blood was allowed to stand at room temperature for 2 h, and serum was obtained by centrifugation at 3000 rpm for 10 minutes. Serum was inactivated in a 56 °C water bath for 30 minutes, cooled on ice, and then sterilized by filtration through a 0.22 μm filter membrane. Finally, serum from the treatment group and the blank control group was mixed, aliquoted in equal volumes, and stored at -80 °C for future use.

### 2.3 Animal models and treatments

A total of 80 male C57BL/6 mice (7 weeks old, 19–22 g) were obtained from Beijing Sibefo Biotechnology Co., Ltd. [license No. SCXK (Jing) 2024-0001]. Mice were maintained under specific pathogen-free (SPF) conditions and acclimatized for one week. All procedures were approved by the Ethics Committee of Inner Mongolia Medical University (Approval No. YKD202404104). Several studies have used intraperitoneal injection of 20% CCl_4_ (typically 1-5 μL/g, twice weekly for 6–8 weeks) to induce liver fibrosis in C57BL/6 mice (24, 32). To minimize potential mortality associated with higher doses (e.g., 5 μL/g) and to ensure model stability and reproducibility, a moderate dosing regimen was selected based on preliminary experiments. In this study, CCl_4_ was diluted in olive oil (1:4, v/v) and administered intraperitoneally at 3 μL/g body weight, twice weekly for 6 weeks, while control mice received olive oil alone. This model is widely validated for inducing hepatic fibrosis (33). Fibrosis progression and successful establishment of stage F3 were confirmed by H&E and Masson staining.

After model validation, mice were randomly assigned into groups (n = 10–15): model control, three QRF-treated groups (1.595, 3.19, and 6.38 g·kg^-^¹), and a silymarin positive control group. From week 7, treatments were administered daily by oral gavage for 6 weeks. CCl_4_ injections were continued once weekly to maintain fibrosis. This induced model and treatment strategy are widely used for antifibrotic evaluation (29). Mice were anesthetized and blood samples were collected and centrifuged to obtain serum for biochemical analysis. Liver and spleen were harvested and weighed. Liver tissues were either fixed for histological analysis or stored at −80 °C for protein and RNA extraction (33, 34). General health status was monitored throughout the experiment (35).

### 2.4 Cell culture, model establishment, and pharmacological interventions

RAW 264.7 murine macrophages (PunoCell, CL-0190) were cultured in a dedicated medium (PunoCell, CM-0190) at 37 °C in a humidified 5% CO_2_ incubator. All experiments used cells from passages 4–8 in the logarithmic growth phase. To evaluate the potential cytotoxicity of drug-containing serum, a Cell Counting Kit-8 (CCK-8; BBI Life Sciences, DCM7122) assay was performed. Cells were seeded in 96-well plates at 1 × 10^5^ cells/well. After adhesion, they were treated with a series of final concentrations of drug-containing serum (2.5%, 5%, 7.5%, 10%, 12.5%, 15%, 20%) for 24 h. Corresponding concentrations of blank serum served as controls. Following incubation, 10 µL of CCK-8 solution was added per well, and plates were incubated for a further 2 h. Absorbance at 450 nm was measured with a microplate reader. The highest concentration that did not cause significant cytotoxicity was selected for subsequent experiments.

To induce pyroptosis, RAW 264.7 cells were seeded in 6-well plates at 5 × 10^5^ cells per well. Then the cells were stimulated with LPS (20 ng/mL) for 4 h, followed by exposure to ATP (5 nM) for 30 min to activate the NLRP3 inflammasome. For the intervention studies, the drug-containing serum (at the concentration determined to be non-cytotoxic) was added to the cells for 1 h following the LPS/ATP stimulation (36). In experiments designed to investigate the role of the NLRP3 inflammasome, the specific agonist nigericin (NIG, MCE, 28380-24-7) was used. NIG (10 µM) was added to the culture 1 h after the administration of the drug-containing serum and incubated for 2 h. Corresponding control groups received equivalent volumes of blank serum or vehicle. After the treatments, cells and supernatants were collected for subsequent analyses.

### 2.5 Measurement of serum biochemical indicators

The serum concentrations of alanine aminotransferase (ALT), aspartate aminotransferase (AST), and alkaline phosphatase (ALP) were quantified using an automatic biochemical analyzer (Mindray BS-350E, China) along with the corresponding reagents (Catalogue Nos.: 140125007, 140225005, and 140324013). Meanwhile, lactate dehydrogenase (LDH) activity was assessed with an assay kit (A020-2-2; Nanjing Jiancheng Bioengineering Institute, China).

### 2.6 Histopathological analysis

After fixation, liver tissues were dehydrated, cleared, and paraffin-embedded. Serial sections (5 μm) were prepared and stained with hematoxylin and eosin (H&E; G1003, Servicebio, China) and Masson’s trichrome (G1006–100 ml, Xi’an Baiswei Biotechnology, China) to assess inflammation and collagen deposition. Histological evaluation was independently performed by two blinded hepatopathologists. Fibrosis was scored using the semi-quantitative SSS system: S = L + P + 2 × (N × W), where L (lobular), P (portal), N (septa number), and W (septa width) were graded; thin septa were assigned W = 0.5. Necroinflammatory activity was evaluated using the Knodell Histological Activity Index (HAI), and fibrosis stage was further assessed using the Metavir system (F0-F4) (6, 37). Discrepancies were resolved by joint review, while minor differences were averaged. For quantitative analysis, ten random fields per section were captured, and collagen-positive areas in Masson-stained sections were measured using Image-Pro Plus 6.0 (Media Cybernetics, USA).

### 2.7 Cytokine and hydroxyproline analysis

Following homogenization and centrifugation of mouse liver tissues, the resultant supernatants were gathered. IL-1β and IL-18 levels were analyzed according to the kit protocols using commercially available ELISA kits (SEKM-0002 and SEKM-0019; Beijing Solarbio Science & Technology Co., Ltd., China). Collagen deposition was assessed by quantifying the hydroxyproline content with a dedicated kit (A030-2-1; Nanjing Jiancheng Bioengineering Institute, China).

### 2.8 Immunohistochemistry

Paraffin-embedded liver tissues (5 mice per group) were sectioned at 4-5 μm for immunohistochemistry. After deparaffinization and rehydration, antigen retrieval was performed in citrate buffer (pH 6.0; ZLI-9065, Zhongshan Jinqiao) using heat treatment. Endogenous peroxidase activity was blocked with 3% H_2_O_2_, and nonspecific binding was prevented with 3% BSA (BioFroxx, 4240GR100). Sections were incubated overnight at 4°C with primary antibodies against α-SMA (14395-1-AP; Proteintech; dilution 1:500) and collagen I (14695-1-AP; Proteintech; 1:500), followed by HRP-conjugated secondary antibody (Maixin Biotech, KIT-5005). Signals were visualized using DAB, followed by hematoxylin counterstaining, dehydration, clearing, and mounting. Positive staining was identified as brown cytoplasmic α-SMA or ECM deposition of collagen I. Stained sections were independently evaluated by two blinded pathologists for staining intensity and area. Quantification was performed using Image-Pro Plus, and data were analyzed as described in the Statistical Analysis section.

### 2.9 Molecular docking

To identify potential active compounds from the traditional Chinese medicine (TCM) formulation, a systematic approach was employed to select small molecules based on specific criteria. These criteria included a minimum threshold for oral bioavailability (OB) of 30% and a drug likeness (DL) of at least 0.18. Their three-dimensional structures were retrieved from the PubChem (https://pubchem.ncbi.nlm.nih.gov) and TCMSP (https://www.tcmsp-e.com/tcmsp.php) databases. Molecular docking was conducted utilizing Discovery Studio software (version 2022). The crystal structure of the target protein, obtained from the Protein Data Bank (PDB, https://www.rcsb.org/), was preprocessed by removing nonstandard residues and heteroatoms, adding hydrogen atoms via the “Clean Protein” module, and optimizing the energy with the “Prepare Protein” tool. Ligands were prepared using the “Prepare Ligands” module. The binding site was defined according to the structural characteristics of the protein via the “From Current Selection” function. the “Dock Ligands (CDOCKER)” protocol was utilized to perform docking calculations. To assess the protein–ligand binding affinity, the binding energy score, referred to as -CDOCKER_ENERGY, was analyzed.

### 2.10 Transcriptome sequencing and data analysis

Liver tissue samples were obtained from three randomly chosen mice from each group (including the blank control, model, and QRF-treated groups) to maintain uniformity in the anatomical site. Total RNA was extracted from every liver sample, and its purity and integrity were confirmed using agarose gel electrophoresis. Following this, the construction of the RNA library and the generation of clusters were completed, followed by paired-end (PE) sequencing conducted on the MGISEQ-T7 platform by MetWare Biotechnology, Inc. Clean reads of high quality were acquired through data filtering and subsequently aligned with the reference genome (*Mus musculus*). After quantifying gene expression, analyses for differential expression, Gene Ontology (GO) and Kyoto Encyclopedia of Genes and Genomes (KEGG) enrichment, and clustering were carried out.

### 2.11 Immunofluorescence

Multiplex immunofluorescence with tyramide signal amplification (TSA) was performed on paraffin-embedded liver sections (5 μm). After deparaffinization (Hongci H-H0101) and rehydration (Sinopharm, 10009218), antigen retrieval was conducted in citrate buffer (Zhongshan Jinqiao, ZLI-9065, pH 6.0) at 95 °C for 20 min. Endogenous peroxidase was quenched with 3% H_2_O_2_, followed by permeabilization with 0.1% Triton X-100 and blocking with 3% BSA (BioFroxx, 4240GR100). Sections were incubated with primary antibodies against F4/80 (Abcam, ab300421; 1:500), iNOS (Abcam, ab178945; 1:500), and Caspase-1 (Proteintech, 22915-1-AP; 1:500), followed by HRP-conjugated secondary antibody (Maixin, KIT-5005). TSA detection was performed using fluorophores (AAT Bioquest: Fluorescein, 11062; CY3, 11065; CY5, 11066), with HRP inactivation between cycles. Nuclei were counterstained with DAPI (Beyotime, P0131). Images were acquired using an Olympus fluorescence microscope, and positive signals were quantified using threshold-based segmentation in Image-Pro Plus 6.0.

### 2.12 Terminal deoxynucleotidyl transferase dUTP nick end labeling assay

TUNEL staining was performed on liver sections embedded in paraffin, with a commercial kit (KGA1406-100; Jiangsu KGI Biotechnology). Briefly, after the processes of deparaffinization and rehydration, the sections underwent treatment with proteinase K at 37 °C for 30 minutes. This step was succeeded by an incubation period with a TdT enzyme reaction mixture at the same temperature for one hour, followed by an additional 30 minutes with streptavidin-FITC. Finally, nuclei were counterstained with DAPI. Fluorescent images were captured at 200 × magnification with an Olympus microscope, and TUNEL-positive cells were quantified with Image-Pro Plus 6.0.

### 2.13 Quantitative real-time PCR

Total RNA was isolated from liver tissues, utilizing six biological replicates, through the RNA Easy Fast Kit (Tiangen Biotech, China). Following extraction, 1 μg of RNA underwent reverse transcription with the HiScript-II Reverse Transcriptase Kit from the same company. Quantitative PCR was then performed in technical triplicates with SYBR^®^ Green Pro Taq HS Premix (Tiangen Biotech) on a real-time PCR system. The thermal profile included an initial 5-min denaturation at 95 °C, followed by 40 cycles of 95 °C for 15 sec and 60 °C for 30 sec. *Gapdh* served as the endogenous control for normalizing gene expression, and the relative mRNA abundance was determined using the 2^-ΔΔCt method. All primer sequences, which are listed in **Table 1**, were designed and synthesized by Sangon Biotech (Shanghai, China).

**Table 1:** The primers used in this study.

Gene name	Forward (5’-3’)	Reverse (5’-3’)
*GAPDH*	GGCATGGACTGTGGTCATGAG	TGCACCACCAACTGCTTAGC
*Col1a1-1*	CGATGGATTCCCGTTCGAGT	GAGGCCTCGGTGGACATTAG
*α-SMA*	GCCATCTTTCATTGGGATGGA	CCCCTGACAGGACGTTGTTA
*Timp1*	AGAGACACACCAGAGATACCA	TATGACCAGGTCCGAGTTGC
*IL-1β*	TGCCACCTTTTGACAGTGATG	TGATGTGCTGCTGCGAGATT
*IL-18*	TCCAACTGCAGACTGGCAC	CTGATGCTGGAGGTTGCAGA
*Caspase-1*	CTATGGACAAGGCACGGGAC	TCAGCTGATGGAGCTGATTGA
*ASC*	TGACAGTGCAACTGCGAGAA	GTGAGCTCCAAGCCATACGA
*GSDMD*	AGTGCTCCAGAACCAGAACC	CCTTCTCCCATGCCTGACAA
*NLRP3*	CAAGGCTGCTATCTGGAGGAA	TGCAACGGACACTCGTCATC
*F4/80*	TGACTCACCTTGTGGTCCTAA	CTTCCCAGAATCCAGTCTTTCC
*iNOS*	CAGGGCCACCTCTACATTTG	TGCCCCATAGGAAAAGACTG
*CD206*	GTGGAGTGATGGAACCCCAG	CTGTCCGCCCAGTATCCATC
*TNF-α*	TAGCCCACGTCGTAGCAAAC	ACAAGGTACAACCCATCGGC
*Ly6C*	GCAGTGCTACGAGTGCTATGG	ACTGACGGGTCTTTAGTTTCCTT
*IL-10*	TGCACTACCAAAGCCACAAG	TCAGTAAGAGCAGGCAGCAT

### 2.14 Western blot analysis

Protein was extracted from liver tissues using RIPA lysis buffer (Beyotime, P0013C) supplemented with 1 mM PMSF (Beyotime, P1082) and a 1× protease/phosphatase inhibitor cocktail (Beyotime, P0015). After incubation on ice for 30 min, samples were centrifuged at 12,000fu for 15 min at 4 °C, and the supernatant was collected. Protein concentration was determined using a BCA assay kit (Beyotime, P0009). Equal amounts of protein were separated by SDS-PAGE and transferred to nitrocellulose membranes. After blocking with 5% skim milk for 2 h at room temperature, membranes were incubated overnight at 4 °C with primary antibodies against GSDMD (Huaan Biotechnology, HA721144; 1:2000), ASC (10500-1-AP; 1:2000), Caspase-1 (22915-1-AP; 1:2000), NLRP3 (68102-1-Ig; 1:2000), IL-1β (26048-1-AP; 1:2000), IL-18 (10663-1-AP; 1:2000), and GAPDH (60004-1-Ig; 1:10000). After TBST washing, membranes were incubated with HRP-conjugated goat anti-rabbit secondary antibody (ZSGB-BIO, ZB-2301; 1:10000) for 1 h at room temperature. Protein bands were visualized using enhanced chemiluminescence (ECL) (Meilunbio) and quantified with ImageJ software (National Institutes of Health, USA). Target protein levels were normalized to GAPDH as an internal control.

### 2.15 Transmission electron microscopy

For ultrastructural analysis, cell pellets were fixed in 2.5% glutaraldehyde in 0.1 M phosphate buffer (pH 7.4) at room temperature for 2 h, followed by post-fixation in 1% osmium tetroxide for 2 h. After dehydration through a graded ethanol-acetone series, the samples were embedded in epoxy resin. Ultrathin sections (60–80 nm) were cut with an ultramicrotome, stained with uranyl acetate and lead citrate, and examined under a transmission electron microscope (JEM 1400, JEOL, Japan). Images were captured to evaluate morphological changes indicative of pyroptosis, such as cell swelling, membrane pores, and organelle disruption.

### 2.16 Statistical analysis

Data are presented as mean ± standard deviation (SD), and statistical significance was set at p < 0.05. Normality of each dataset was first assessed using the Shapiro-Wilk test. Comparisons between two groups were performed using Student’s t-test. For multiple group comparisons, one-way analysis of variance (ANOVA) followed by Bonferroni *post hoc* test was used if data were normally distributed and exhibited homogeneity of variance (Levene’s test); otherwise, the nonparametric Kruskal-Wallis test followed by Dunn’s *post hoc* test was applied. All *post hoc* pairwise comparisons were adjusted for multiple testing using the appropriate correction (Bonferroni or Dunn method). Statistical analyses were conducted using GraphPad Prism 7 (GraphPad Software Inc., La Jolla, CA, USA) and IBM SPSS Statistics 27 (IBM Corporation, Armonk, NY, USA).

## 3 Results

### 3.1 Pharmacodynamic assessment of QRF at different doses in mice with stage F3 liver fibrosis

#### 3.1.1 QRF dose-dependently improves general health condition in fibrotic mice

**Figure 1:**
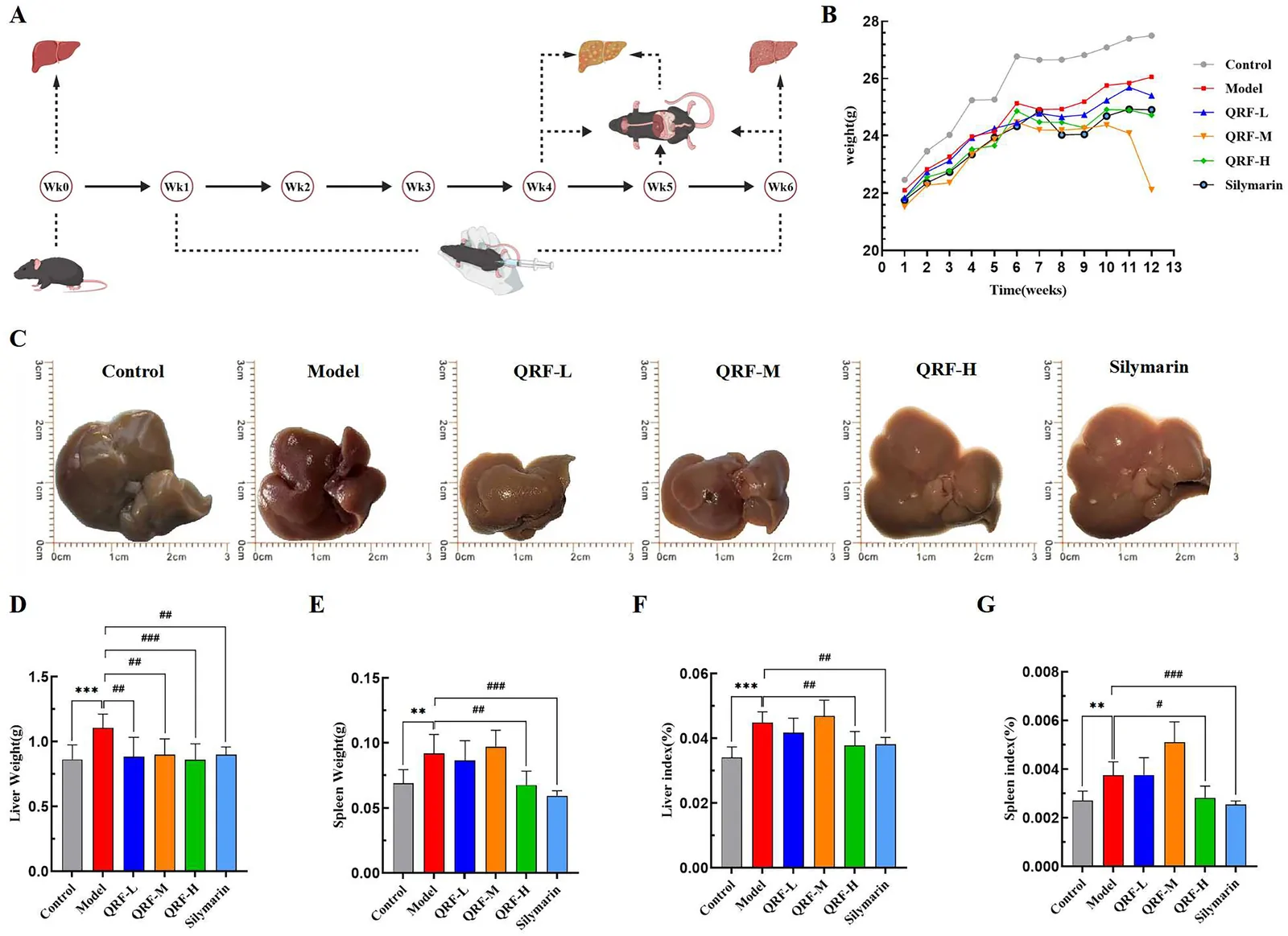
Effects of QRF on body weight and organ indices in mice with CCl₄-induced stage F3 liver fibrosis. **(A)** Experimental timeline: 1-week acclimatization followed by 6 weeks of CCl₄ administration. **(B)** Body weight changes. **(C)** Typical images depicting liver structure. **(D, E)** Liver and spleen weights. **(F, G)** Liver and spleen indices (organ weight/body weight ×100%). Data are presented as mean ± SD (n = 9 mice per group). **p < 0.01, ***p < 0.001 vs. Control; ^#^p < 0.05, ^##^p < 0.01, ^###^p < 0.001 vs. Model. QRF groups: low-, medium-, and high-dose (QRF-L/M/H). WK, week.

A stage F3 liver fibrosis model was established using CCl_4_ (**Figure 1A**), with detailed evaluation provided in the Supplementary Material (**Supplementary Figures S1**, **S2**; **Table S1**). Following 6 weeks of QRF treatment, overall condition improved in a dose-dependent manner compared with the model group. Notably, the high-dose QRF group exhibited effects comparable to Silymarin, with marked improvements in activity and fur condition. In contrast, the medium-dose group showed reduced activity and poorer fur quality from week 10, while the low-dose group demonstrated moderate improvement. During the intervention period (weeks 7–12), body weight in the control group increased steadily, albeit at a slower rate. In contrast, all other groups exhibited significantly lower body weights after week 6 (p < 0.05 to p < 0.001), followed by an initial decline and partial recovery. The medium-dose group showed a sustained decrease from week 10, resulting in the lowest body weight at the endpoint (P < 0.001). Of particular note, the high-dose QRF group exhibited a transient decline at week 8, followed by recovery, with a slight reduction at the end of the experiment (**Figure 1B**).

Grossly, livers in the control group appeared smooth and uniform, whereas those in the model group exhibited granular or nodular changes (**Figure 1C**). These pathological alterations were accompanied by significant increases in liver and spleen wet weights, as well as their respective indices (p < 0.01 to p < 0.001). Treatment with high-dose QRF or silymarin markedly reduced these parameters (p < 0.05 to p < 0.001), with liver morphology approaching normal. In contrast, medium- and low-dose QRF produced only a partial effect, as evidenced by reduced liver weight (p < 0.01) without significant improvement in spleen weight or organ indices, and persistent granular lesions in the medium-dose group (**Figures 1D–G**).

Collectively, these findings indicate that QRF exerts dose-dependent antifibrotic effects, with the high-dose regimen showing the greatest efficacy, supporting its use in subsequent experiments.

#### 3.1.2 QRF dose-dependently alleviates liver fibrosis in F3-stage mice

**Figure 2:**
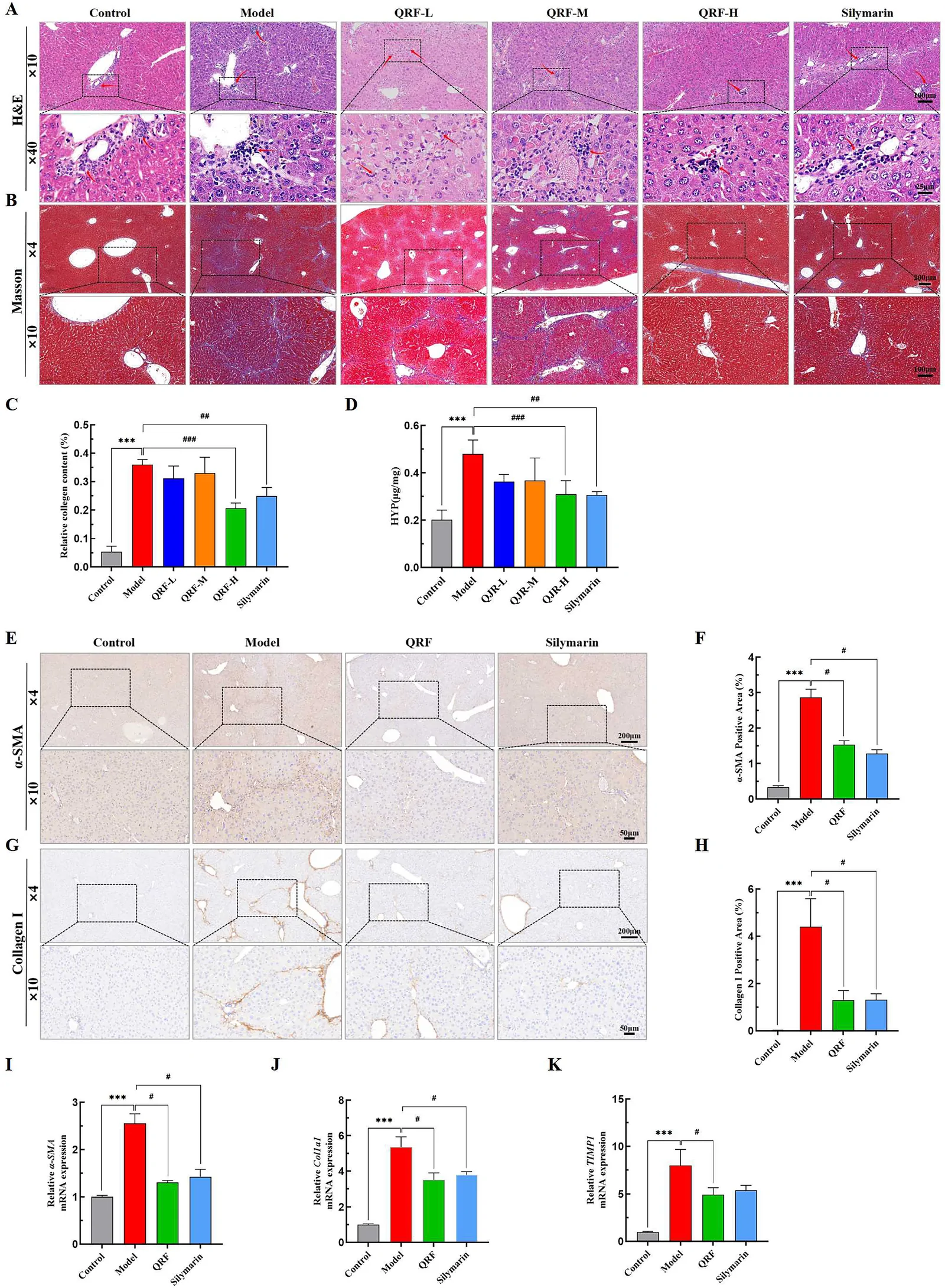
QRF improves CCl_4_-induced liver fibrosis in mice. **(A)** H&E staining (scale bars: 100 μm at ×10; 25 μm at ×40). **(B)** Masson’s trichrome staining (scale bars: 200 μm at ×4; 100 μm at ×10), accompanied by **(C)** Quantitative evaluation of collagen volume fraction. **(D)** Hepatic hydroxyproline (Hyp) levels. **(E, G)** Immunohistochemistry for **(E)** α-SMA and **(G)** Collagen I (scale bars: 200 μm at ×4; 50 μm at ×10), alongside **(F, H)** corresponding integrated optical density (IOD) assessments. **(I–K)** qRT–PCR analysis of **(I)** *α-SMA*, **(J)** *Col1a1*, and **(K)** *Timp-*1 mRNA levels. Data are presented as mean ± SD (n=4–6 mice per group). ***p < 0.001 *vs*. Control; ^#^p < 0.05, ^##^p < 0.01, ^###^p < 0.001 *vs.* Model.

To evaluate the dose-dependent efficacy of QRF in stage F3 liver fibrosis, integrated histopathological, biochemical, and molecular analyses were performed. H&E and Masson staining (**Figures 2A, B**) showed intact hepatic architecture with minimal collagen deposition in the untreated group, whereas the model group exhibited marked inflammatory infiltration, necrosis, and extensive fibrotic septa. QRF exerted a clear dose-dependent antifibrotic effect, with high-dose treatment ameliorated fibrosis, reducing the stage from F3 to F2 in 90% of mice and preventing progression to F4, while medium- and low-dose groups showed only 10% showing stage improvement (**Supplementary Figures S3**, **S4**; **Table S2**). Notably, the effect of high-dose QRF was comparable to silymarin (**Figure 2B**).

Quantitative Masson analysis confirmed significantly increased collagen deposition in the model group (p < 0.001), which was markedly reduced by high-dose QRF (**Figure 2C**, p < 0.001). Consistently, hepatic hydroxyproline levels were significantly decreased (**Figure 2D**, p < 0.001). Immunohistochemistry further demonstrated reduced α-SMA and collagen I expression (**Figures 2E–H**), indicating inhibition of HSCs activation and ECM accumulation. qRT-PCR analysis indicated that high-dose QRF significantly reduced mRNA levels of key fibrosis-related genes, including *α-SMA*, *Col1a1*, and tissue inhibitor of *metalloproteinase 1* (*Timp1*) (**Figures 2I–K**). These results indicate that high-dose QRF suppressed HSCs activation (α-SMA) and ECM deposition (*Col1a1*), while simultaneously promoting ECM degradation through downregulation of *Timp1* at the transcriptional level, collectively confirming its anti-fibrotic effect.

#### 3.1.3 QRF dose-dependently attenuates liver dysfunction in fibrotic mice

**Figure 3:**
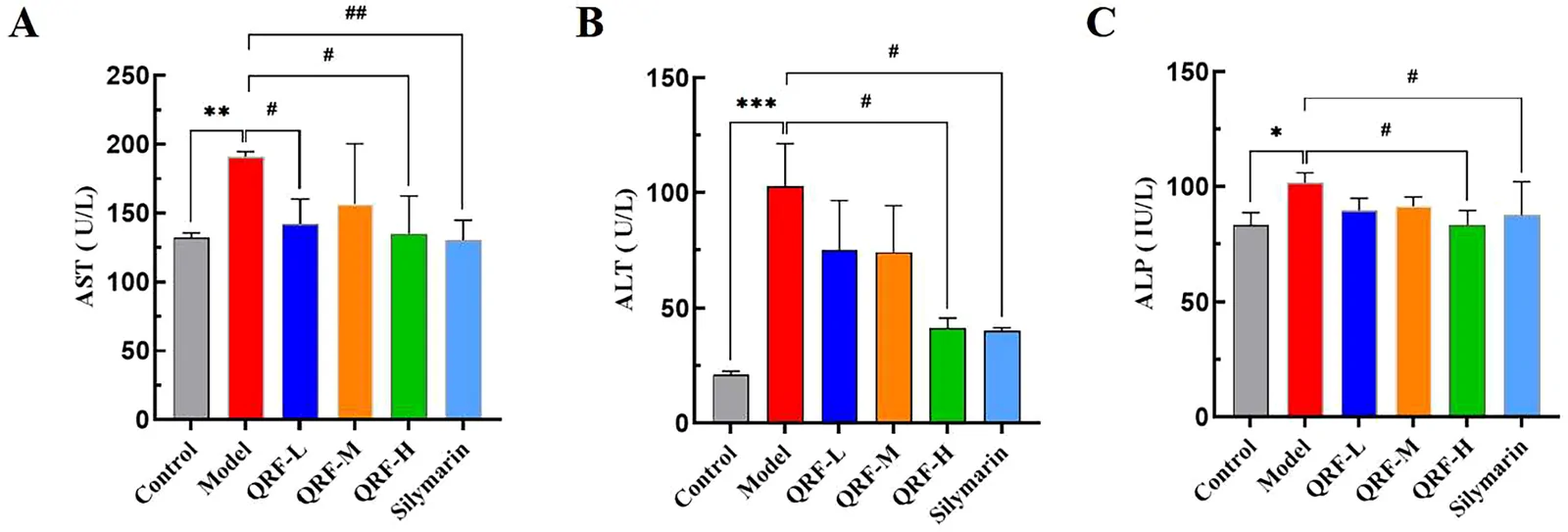
Impact of QRF on serum biomarkers in mice with stage F3 liver fibrosis. Following a treatment period of 6 weeks, the serum levels of **(A)** AST, **(B)** ALT, **(C)** ALP were evaluated. Data are presented as mean ± SD (n=3 mice per group). *p < 0.05, **p < 0.01, ***p < 0.001 vs. Control; ^#^p < 0.05, ^##^p < 0.01 vs. Model.

To assess how QRF affects liver function in mice with stage F3 fibrosis in a dose-dependent way, serum concentrations of AST, ALT and ALP, were evaluated. In comparison to the control group, the model group treated with CCl_4_ demonstrated significantly elevated concentrations of all markers (p < 0.05 to p < 0.001). intervention with QRF led to improvements that depended on the dosage: the group receiving a low dose significantly reduced AST levels (p < 0.05; **Figure 3A**), while the medium-dose group displayed downward trends in ALT and ALP levels that lacked statistical significance (**Figures 3B, C**). In contrast, the high-dose group treated with QRF exhibited substantial decreases in all measured parameters (p < 0.05), demonstrating effects similar to those observed with the positive control drug silymarin.

### 3.2 Mechanism of the QRF against stage F3 liver fibrosis

#### 3.2.1 Molecular docking of QRF active components with pyroptosis-related proteins

**Figure 4:**
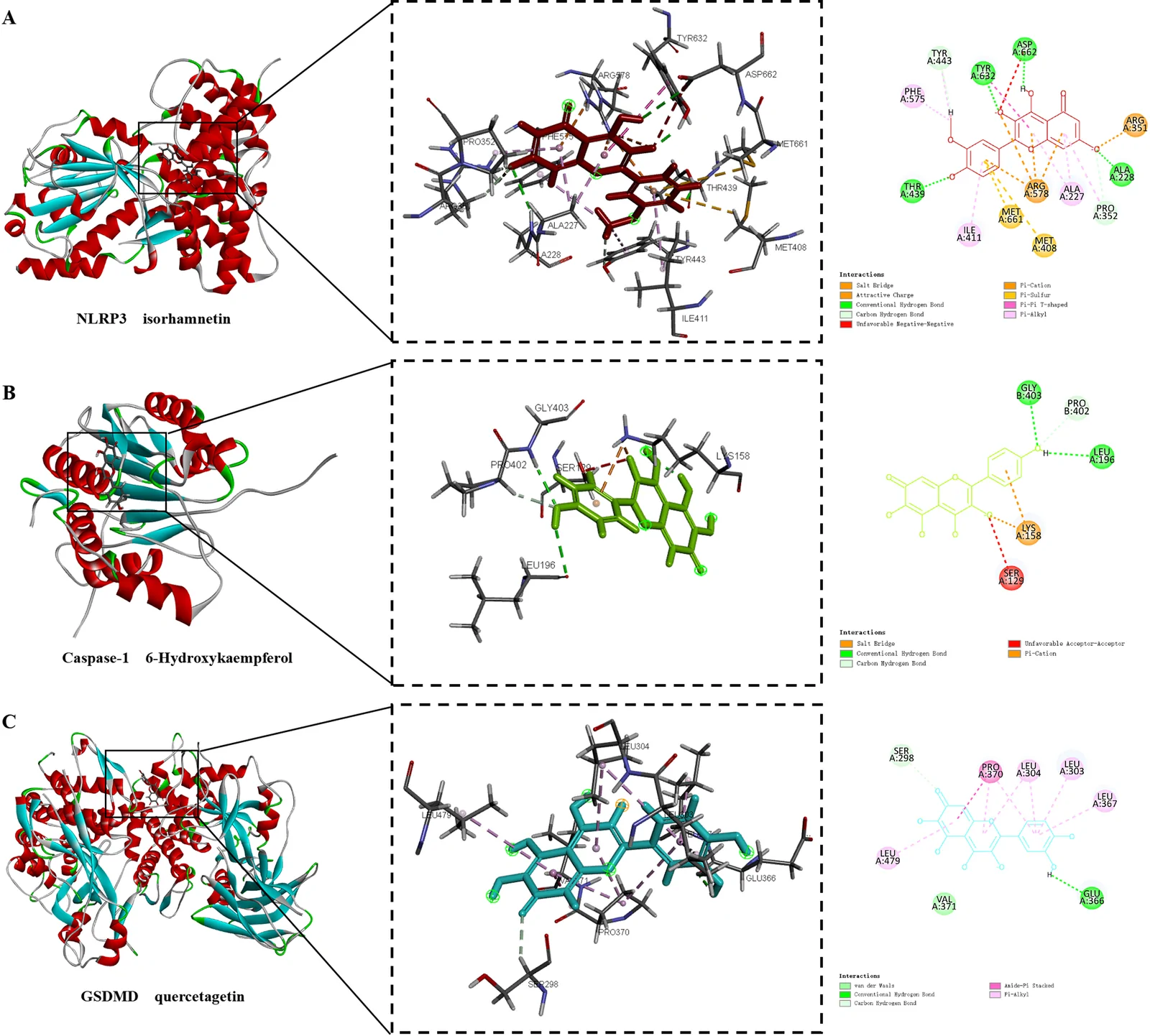
Analysis of molecular docking for QRF interacting with NLRP3, Caspase-1, and GSDMD. **(A–C)** Results from the molecular docking study showcasing the interactions between the key active constituents of QRF and **(A)** NLRP3, **(B)** Caspase-1, and **(C)** GSDMD.

To clarify the molecular mechanisms underlying the anti-fibrotic effects of QRF via modulation of the NLRP3 inflammasome-mediated pyroptosis pathway, we performed molecular docking analyses of its principal bioactive components with key proteins in the NLRP3/Caspase-1/GSDMD pathway. Docking results demonstrated strong binding interactions: isorhamnetin exhibited the highest affinity for NLRP3 (PDB ID:7ALV; -CDOCKER_ENERGY: -59.3506 kcal/mol), followed by 6-hydroxykaempferol with Caspase-1 (PDB ID:1RWK; -CDOCKER_ENERGY: -30.5207 kcal/mol) and quercitrin with GSDMD (PDB ID:7Z1X; -CDOCKER_ENERGY: -29.9735 kcal/mol). The notably low CDOCKER energies, particularly for the isorhamnetin-NLRP3 complex, indicate stable and thermodynamically favorable binding. Detailed analysis of the binding modes revealed distinct interaction patterns for each compound-protein pair, including hydrogen bonds and hydrophobic contacts at their respective active sites (**Figure 4**). These computational findings provide evidence that the active constituents of QRF can target multiple nodes of the NLRP3 inflammasome pathway, potentially inhibiting pyroptosis and mitigating hepatic fibrosis. Collectively, these results establish a basis for elucidating the anti-fibrotic mechanisms of QRF through modulation of pyroptosis.

#### 3.2.2 Transcriptomic profiling indicates NOD-like receptor pathway as a key target of QRF

**Figure 5:**
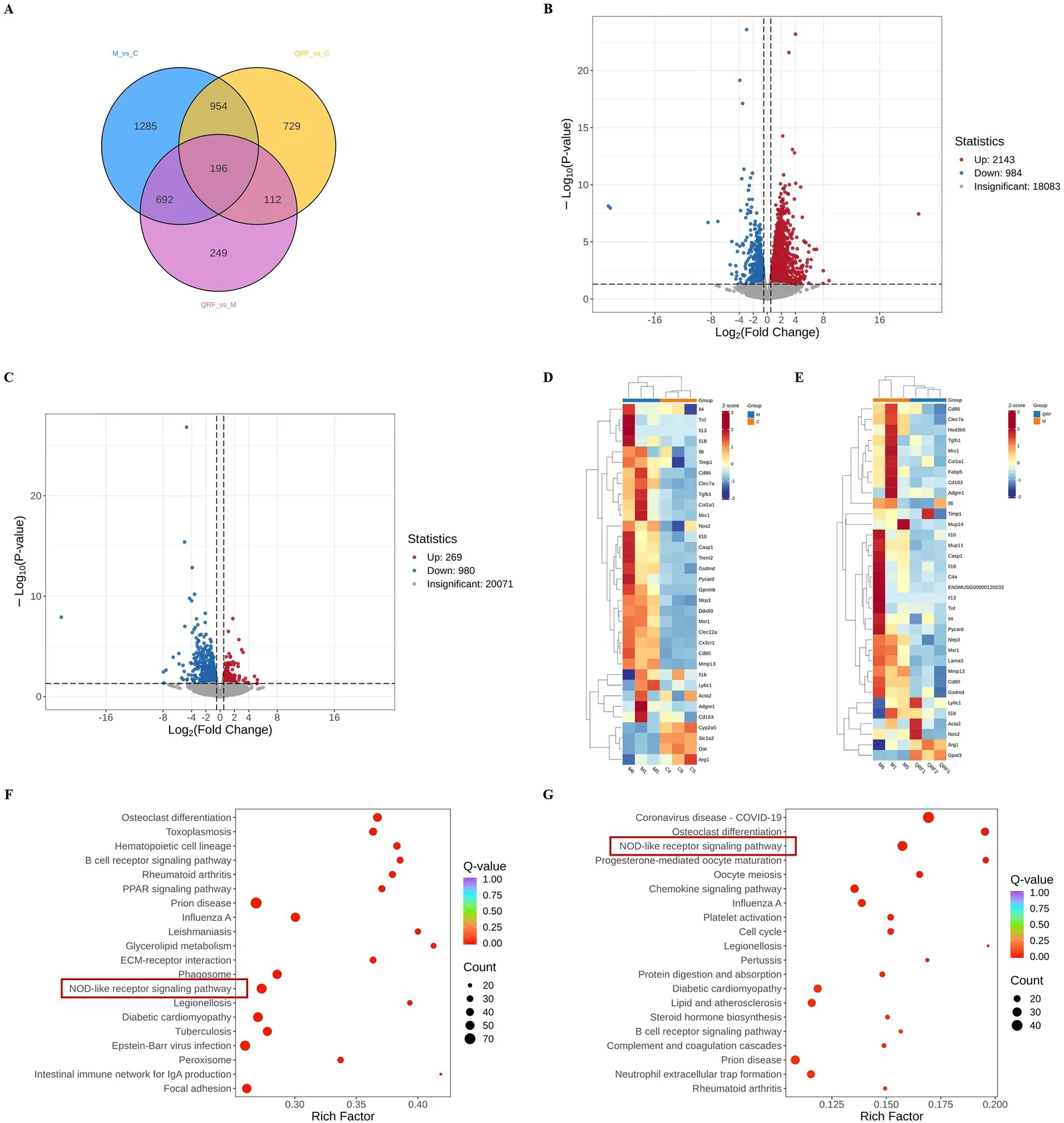
Transcriptomic analysis illustrating the impact of QRF intervention on liver fibrosis at stage F3 induced by CCl_4_. **(A)** A Venn diagram displaying differentially expressed genes (DEGs) from the specified comparisons. **(B, C)** Volcano plots demonstrating DEGs between the model and control **(B)** and between the QRF and model groups **(C)**, respectively. **(D, E)** Heatmaps showcasing the most significant DEGs along with a selection of genes related to macrophage polarization, liver fibrosis, and pyroptosis from the comparisons illustrated in **(B, C)**, respectively. **(F, G)** Scatter plots of KEGG pathway enrichment for DEGs from the comparisons in **(B, C)**, respectively.

To investigate the transcriptomic regulatory mechanisms of QRF in liver fibrosis, eukaryotic transcriptome sequencing was conducted. Our analysis revealed 4,217 differentially expressed genes (DEGs), of which 196 core DEGs were shared across the three comparison groups (**Figure 5A**). Compared with control group, the fibrosis model exhibited 214 upregulated and 984 downregulated genes, whereas QRF treatment reversed these trends, with 269 upregulated and 980 downregulated genes (**Figures 5B, C**). Cluster heatmaps highlighted that pyroptosis-related genes (Nlrp3, Pycard, Casp1, Gsdmd), fibrosis markers (Col1a1, Mmp13, IL-18), and M1 macrophage markers (CD80, CD86) were markedly elevated in the model group, but significantly reduced following QRF treatment (**Figure 5D**). Conversely, the M2 macrophage marker Arg1, suppressed in the model group, was restored by QRF (**Figure 5E**). KEGG enrichment analysis revealed that DEGs in both model *vs*. control and QRF *vs.* model comparisons were prominently enriched in the NOD-like receptor signaling pathway (**Figures 5F, G**). Notably, this pathway, particularly through NLRP3 inflammasome activation, regulates pyroptosis *via* Caspase-1 mediated GSDMD cleavage, representing a key mechanism underlying the antifibrotic effects of QRF.

#### 3.2.3 High-dose QRF inhibits M1 macrophage-associated pyroptosis and promotes M2 polarization

**Figure 6:**
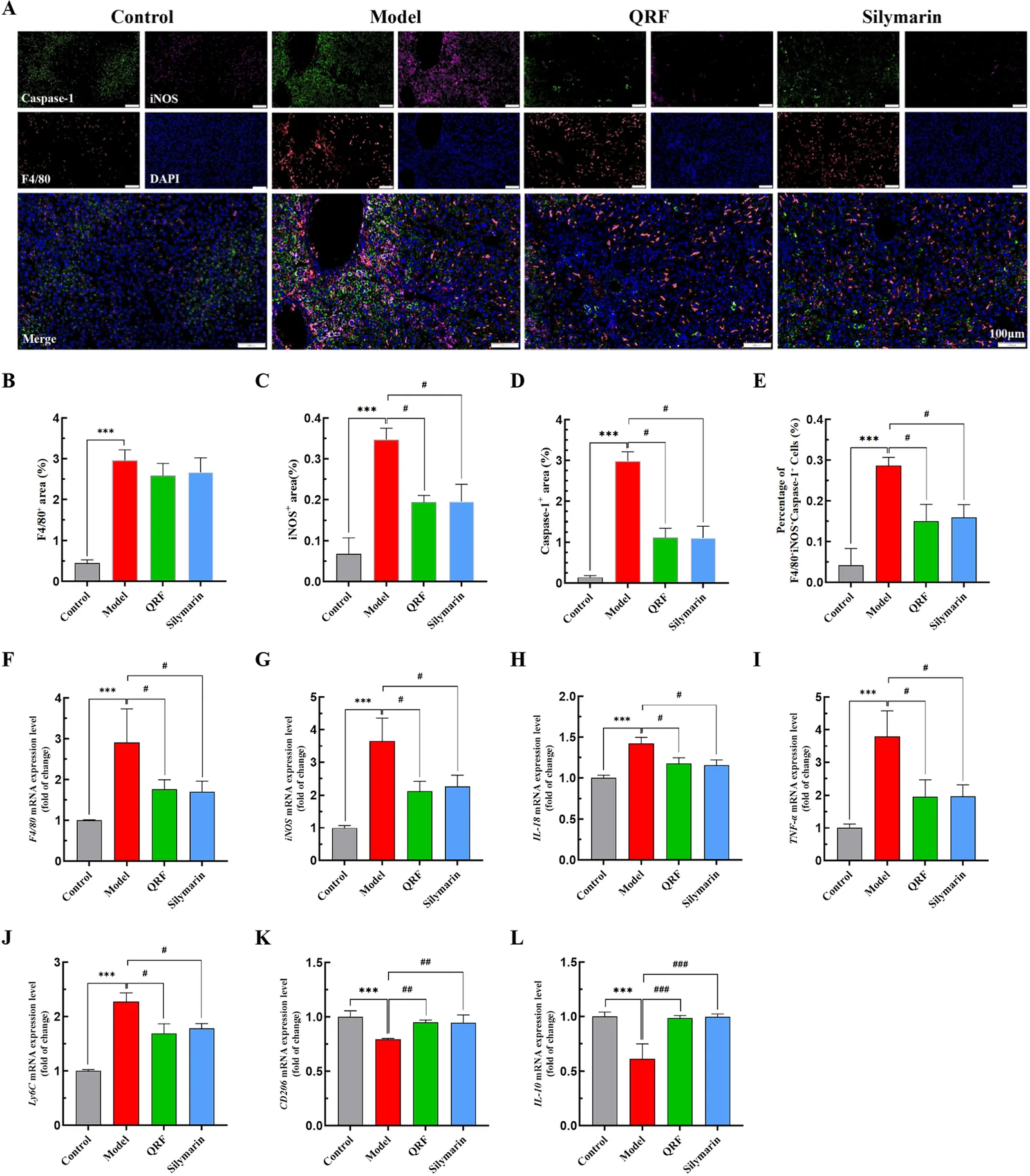
QRF modulates M1/M2 macrophage polarization and pyroptosis in Stage F3 liver fibrosis. **(A)** Immunofluorescence of liver sections showing F4/80 (red, macrophages), iNOS (purple, M1), Caspase-1 (green, pyroptosis), and DAPI-stained nuclei (blue). (20×; scale bar: 100 µm). **(B–E)** Analysis of **(B)** F4/80^+^, **(C)** iNOS^+^, **(D)** Caspase-1^+^ regions, and **(E)** F4/80^+^iNOS^+^Caspase-1^+^ triple-positive cells (n = 5 mice per group). **(F–L)** qRT–PCR of macrophage polarization markers normalized to Gapdh. **(F–J**) M1 markers: Emr1 (F4/80), Nos2 (iNOS), Il18, Tnf, and Ly6c. **(K, L)** M2 markers: Mrc1 (CD206) and Il10. Data are presented as mean ± SD (n = 6 mice per group for M1 markers; n = 3 mice per group for M2 markers). ***p < 0.001 *vs.* Control; ^#^p < 0.05, ^##^p < 0.01, ^###^p < 0.001 *vs.* Model.

To investigate macrophage pyroptosis and phenotypic changes in liver tissues, we performed triple immunofluorescence staining. Compared with controls, the fibrosis model exhibited markedly increased fluorescence of the macrophage marker F4/80, the M1 marker iNOS, and the inflammasome protein Caspase-1 (**Figure 6A**). Colocalization analysis revealed significant overlap between iNOS and Caspase-1, with substantially larger colocalization regions in the model group (**Figures 6B–E**). Consistently, qRT-PCR demonstrated elevated mRNA levels of F4/80, iNOS, IL-18, and Ly6C (all p < 0.001; **Figures 6F–J**), whereas M2-related genes CD206 and IL-10 were downregulated (p < 0.001; **Figures 6K, L**). Collectively, compared to controls, the model group showed enhanced macrophage infiltration, increased pro-inflammatory M1 markers, decreased anti-inflammatory M2 markers, and elevated inflammasome activation and pyroptosis. These indicated that F3-stage is accompanied by macrophage pyroptosis and M2-to-M1 polarization phenotypic shift. Following drug intervention, F4/80 protein levels were unchanged in both high-dose QRF and silymarin groups relative to the model. In contrast, iNOS and Caspase-1 protein levels were significantly reduced (**Figures 6C, D**). qRT-PCR confirmed that treatment markedly decreased iNOS, IL-18, TNF-α, and Ly6C mRNA levels (p < 0.05), while CD206 and IL-10 expression was significantly increased ((p < 0.01 to p < 0.001; **Figures 6G–L**). These suggested that QRF intervention suppressed M1-associated inflammatory and pyroptosis-related signaling while promoting a shift toward the M2 polarization phenotype. This indicated that QRF alleviates liver fibrosis by inhibiting macrophage pyroptosis and M1 polarization.

#### 3.2.4 High-dose QRF suppresses the NLRP3/Caspase-1/GSDMD pyroptosis pathway

**Figure 7:**
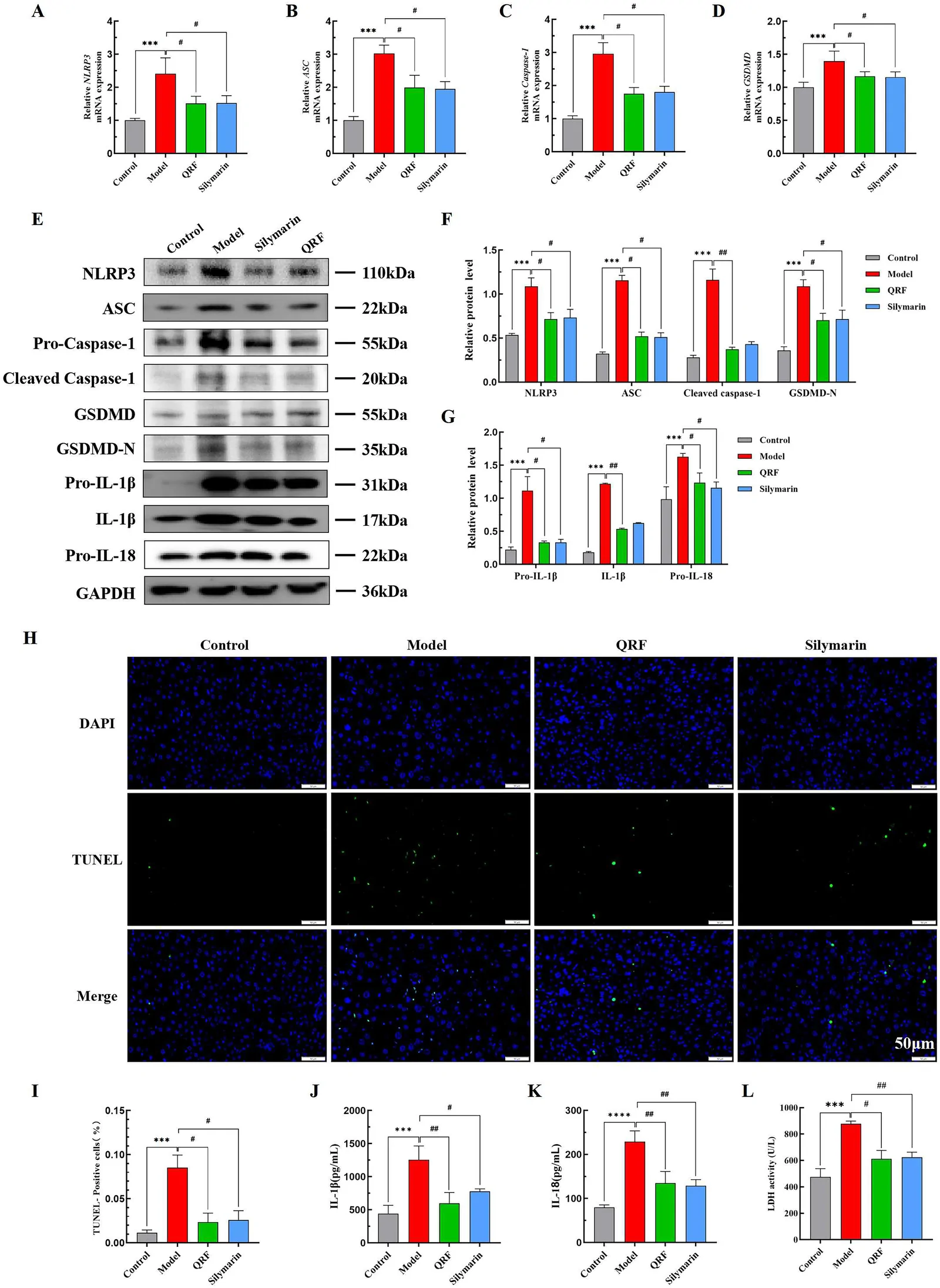
QRF alleviates inflammation, pyroptosis, and fibrosisin stage F3 liver fibrosis. **(A–D)** qRT–PCR analysis of *NLRP3* inflammasome-related gene: *Nlrp3*, *Caspase-1*, *ASC*, and *GSDMD* (normalized to *Gapdh*). **(E)** Representative Western blots ofNLRP3, Pro-Caspase-1, cleaved Caspase-1 (p20), ASC, GSDMD, GSDMD-N, Pro-IL-1β, mature IL-1β, and Pro-IL-18. GAPDH asloading control. **(F, G)** Densitometric analysis of cleaved Caspase-1(p20), ASC, GSDMD-N, Pro-IL-1β, mature IL-1β, and Pro-IL-18. **(H)** TUNEL staining (green) in liver sections; nuclei counterstainedwith DAPI (blue). (20bl scale bar: 50 mm). **(I)** Quantification ofTUNEL-positive area. **(J, K)** Hepatic IL-1β and IL-18 levels measured by ELISA. **(L)** LDH levels in supernatants. Data are presentedas mean ± SD (n = 3–6 mice per group). ***p < 0.001, ****p < 0.0001 *vs.* Control; ^#^p < 0.05, ^##^p < 0.01 *vs*. Model.

To determine whether QRF alleviates liver fibrosis *via* modulation of the classical NLRP3/Caspase-1 mediated pyroptosis pathway, we systematically examined the expression and activation of key pathway components. qRT-PCR analysis revealed that, compared to controls, the mRNA levels of NLRP3, Caspase-1, ASC, and GSDMD were significantly upregulated in the livers of model mice, whereas QRF intervention effectively reversed these changes (p < 0.05; **Figures 7A–D**). At the protein level, Western blot confirmed pathway activation, with NLRP3, cleaved Caspase-1 (p20), ASC, and the N-terminal fragment of GSDMD (GSDMD-N) markedly elevated in the model group (p < 0.001; **Figures 7E, F**). Similarly, both precursors (Pro-IL-1β, Pro-IL-18) and the mature cytokine cleaved IL-1β were significantly increased (P < 0.001; **Figures 7E, G**), and QRF treatment effectively suppressed their abnormal accumulation and activation (p < 0.05; **Figures 7E–G**). A significant increase in TUNEL-positive cells was observed in the model group by TUNEL staining, and this signal was markedly reduced upon QRF treatment. (p < 0.05; **Figures 7H,I**). Consistently, ELISA analysis showed elevated levels of mature IL-1β and IL-18 in liver homogenates from the model group, which were suppressed by QRF treatment (p < 0.05; **Figures 7J, K**). Lactate dehydrogenase (LDH) release in the supernatant displayed a similar trend (**Figure 7L**). These results demonstrated that QRF inhibited NLRP3/Caspase-1/GSDMD-mediated pyroptosis in fibrotic livers at multiple levels, including transcription, protein activation, cell death, and inflammatory mediator release.

### 3.3 *In vitro* functional validation of QRF in NLRP3-mediated macrophage pyroptosis

#### 3.3.1 QRF-containing serum attenuates macrophage pyroptosis as revealed by TEM

**Figure 8:**
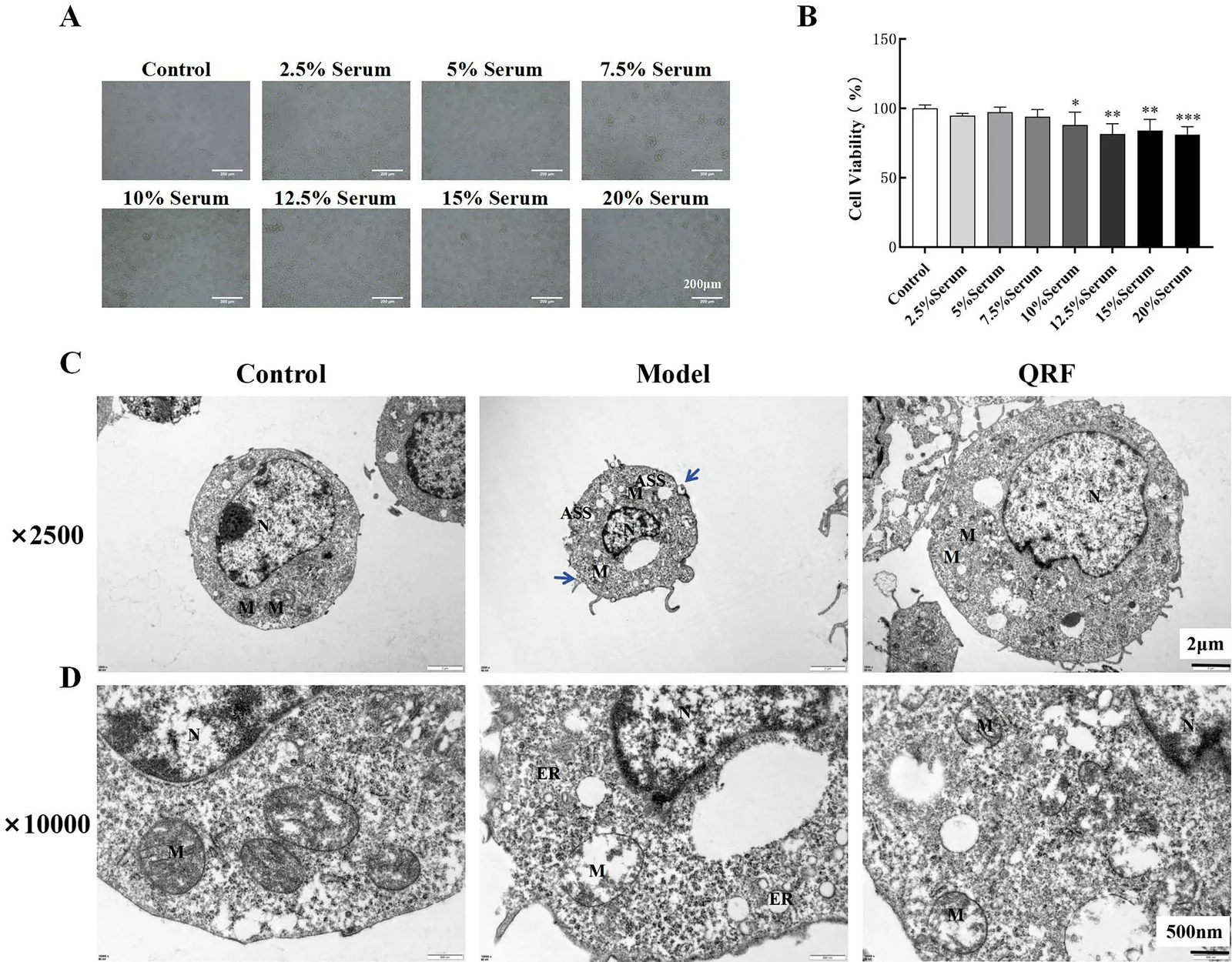
Effects of QRF-containing serum on RAW 264.7 macrophages: non-toxic concentration and ultrastructure. **(A)** Representative morphology of cells treated with different concentrations of QRF-containing serum for 24 h (10 h scale bar: 200 μm). **(B)** Cell viability assessed by CCK-8, with the highest non-toxic concentration selected for subsequent experiments. **(C, D)** TEM images showing cellular ultrastructure: low magnification (**(C)**, 2500if 2 µm) and high magnification (**(D)**, 10000fi 500 nm). The blue arrows indicate multiple ruptures of the macrophage plasma membrane. *p < 0.05, **p < 0.01, ***p < 0.001 *vs.* Control.

LPS/ATP-induced pyroptosis model was established in RAW 264.7 macrophages. CCK-8 assays were performed to evaluate the effect of QRF-containing serum on the viability of the cells. The results showed that, compared with serum-free controls at equivalent concentrations, 2.5%, 5%, and 7.5% QRF-containing serum had no significant impact on cell viability after 24 h of incubation (p > 0.05; **Figures 8A,B**). Therefore, 7.5% serum was selected for subsequent experiments. Transmission electron microscopy (TEM) provided direct morphological evidence of QRF’s inhibitory effect on macrophage pyroptosis (**Figures 8C, D**). Cells in the control group exhibited normal morphology, with regular nuclei (N), intact mitochondria (M), and preserved plasma membranes. In contrast, macrophages in the model group displayed classical pyroptotic features, including cellular swelling, irregular nuclei, severely swollen and vacuolated mitochondria, dilated endoplasmic reticulum (ER), and occasional autophagolysosomes (ASS), with multiple plasma membrane ruptures (blue arrows in **Figure 8D**) indicative of osmotic lysis. Notably, QRF treatment preserved cellular morphology, improved nuclear integrity, alleviated mitochondrial swelling and vacuolization, and maintained membrane integrity. These findings indicate that QRF-containing serum effectively protects macrophage membrane integrity and mitigates LPS/ATP-induced pyroptotic damage.

#### 3.3.2 Functional validation of QRF in suppressing NLRP3-mediated macrophage pyroptosis

**Figure 9:**
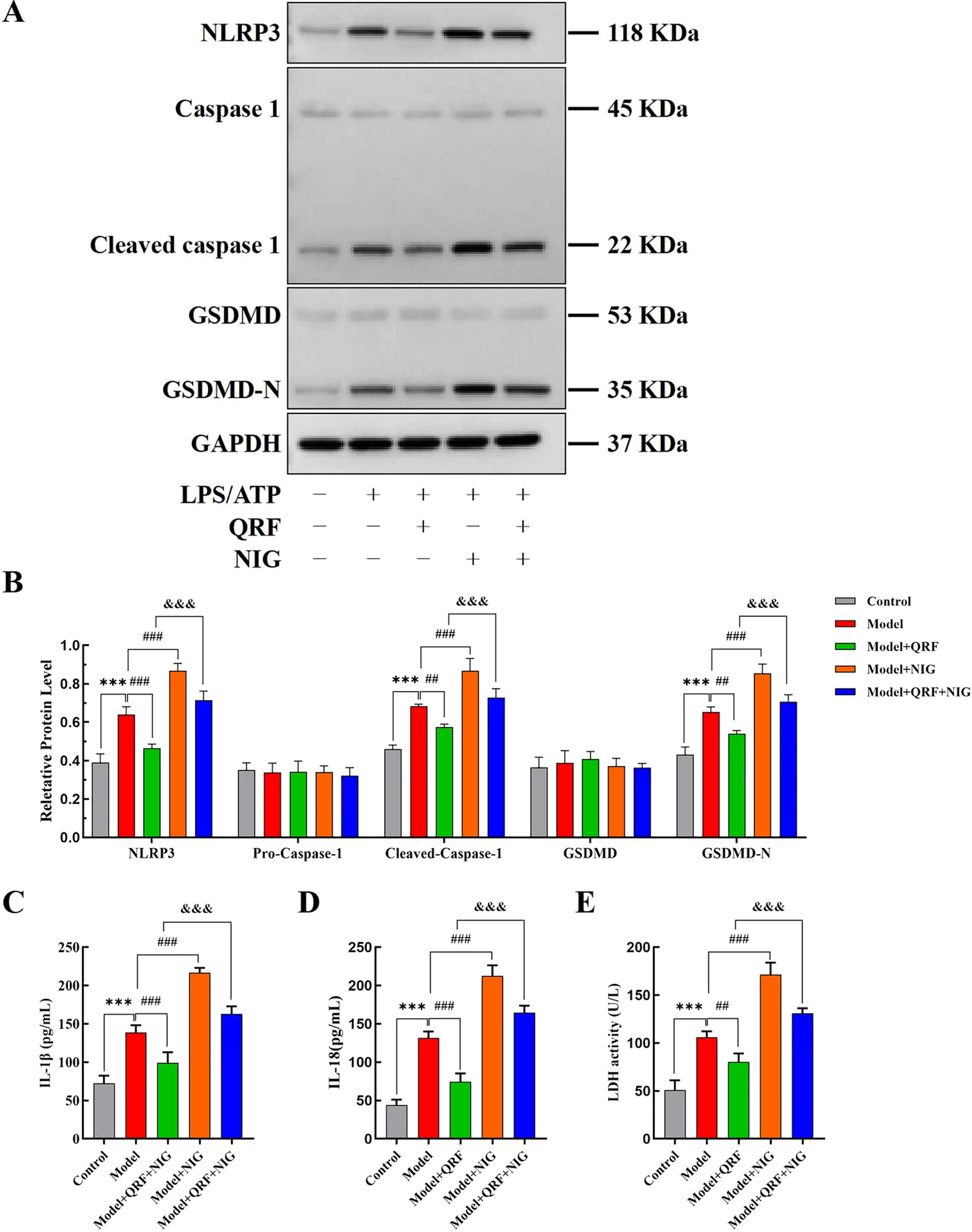
NLRP3 agonist NIG attenuates the inhibitory effect of QRF on macrophage pyroptosis. **(A)** Representative Western blot images of NLRP3, Pro-Caspase-1, cleaved Caspase-1 (p20), full-length GSDMD, and GSDMD-N protein expression in each group. GAPDH served as the loading control. **(B)** Densitometric analysis of band intensity for NLRP3, Pro-Caspase-1, cleaved Caspase-1 (p20), full-length GSDMD, and GSDMD-N. **(C–E)** Concentrations of **(C)** IL-1β and **(D)** IL-18 in cell supernatant, and **(E)** LDH release rate. Data are presented as mean ± SD (n = 3). ***p < 0.001 vs. Control; ^##^p < 0.01, ^###^p < 0.001 vs. Model; ^&&&^p < 0.001 vs. Model + QRF serum group.

LPS/ATP-induced pyroptosis model based on RAW 264.7 macrophages was used to elucidate the anti-pyroptotic mechanisms of QRF-containing serum. Western blot analysis showed that NLRP3, cleaved Caspase-1, and GSDMD-N were markedly upregulated in the model group (p < 0.001), indicating inflammasome activation, whereas QRF treatment significantly reduced their expression (p < 0.01 to 0.001), suggesting inhibition of NLRP3 inflammasome activation. To verify specificity, the NLRP3 agonist nigericin (NIG) was applied. NIG treatment further enhanced pyroptosis-related protein expression compared with the model group (p < 0.001). Notably, co-treatment with QRF and NIG partially reversed its inhibitory effects, resulting in higher levels of NLRP3, cleaved Caspase-1, and GSDMD-N than QRF alone (P < 0.001) (**Figures 9A, B**), supporting NLRP3 as a key target of QRF. Consistently, IL-1β and IL-18 levels in the supernatant were significantly elevated in the model group (p < 0.001) but suppressed by QRF, whereas NIG enhanced their release and attenuated QRF efficacy (**Figures 9C, D**). Similarly, LDH assays showed reduced membrane damage with QRF, while NIG increased LDH leakage (p < 0.001) (**Figure 9E**). Collectively, QRF-containing serum inhibits LPS/ATP-induced macrophage pyroptosis by suppressing NLRP3 inflammasome activation, thereby reducing Caspase-1n,ecedtedD GSDMD cleavage, cytokine release, and membrane damage.

## 4 Discussion

Liver fibrosis represents a critical stage in the progression of chronic liver diseases toward cirrhosis. F3 stage liver fibrosis serves as a pivotal transitional phase that retains the potential for regression and prevents progression to irreversible cirrhosis (38, 39). However, no clinically validated pharmacological or biological therapies are currently available. In this study, traditional Chinese medicine (TCM), characterized by multi-target actions and a favorable safety profile, has gained increasing attention and shown promising anti-fibrotic potential (11–15), particularly in stages F3 and F4 (16, 17). Our group has developed the patented anti-fibrotic formula QRF, whose key components have been supported by pharmacological evidence. For example, polysaccharides and saponins from ‘Astragalus membranaceus’ are known to modulate immune function (40, 41), and the synergistic effects of its constituents likely contribute to its anti-fibrotic efficacy. Our previous RCT research demonstrated that QRF significantly improves fibrosis and clinical symptoms in F3-stage patients (21). However, the pathological mechanisms underlying F3-stage fibrosis and the specific mechanisms of QRF remain unclear. Therefore, this study systematically investigates F3-stage liver fibrosis and evaluates the therapeutic effects of QRF, aiming to provide experimental and mechanistic evidence for targeted intervention at this critical stage.

Although numerous studies have used CCl_4_-induced liver fibrosis models, most reported pathological features correspond to F4-stage or more advanced disease (42, 43). In addition, stage-specific interventions remain scarce, particularly for F3-stage fibrosis. Our team has focused on elucidating the anti-fibrotic effects and mechanisms of TCM formulations and their active components. In this study, we established a stable and reliable CCl_4_-induced F3-stage liver fibrosis mouse model. By week six, the animals exhibited hallmark features of F3-stage fibrosis, including pronounced inflammatory infiltration, extensive fibrous septa formation without pseudolobule development, and elevated serum transaminases. These findings are consistent with the guidelines for the diagnosis and treatment of liver fibrosis and the METAVIR staging criteria (5, 6). During the subsequent six-week treatment period, the model maintained stable F3-stage characteristics while showing a tendency toward progression, thereby effectively recapitulating the natural course of the disease (44).

This study systematically evaluated the dose-dependent anti-fibrotic effects of QRF and demonstrated a clear dose–response relationship. High-dose QRF significantly improved liver function in most mice and markedly attenuated fibrosis, likely by suppressing HSCs activation and ECM deposition. These findings are consistent with previous studies (28, 45), and our prior observations in rat models (19, 20, 22). In contrast, the middle- and low-dose groups showed limited efficacy, with some animals exhibiting disease progression. This may reflect insufficient intrahepatic drug concentration, as lower doses may not achieve concentrations adequate to effectively inhibit HSCs activation, potentially due to first-pass metabolism and off-target distribution, thereby failing to halt fibrosis progression. These results are in line with established dose–response principles in liver-targeted therapies (46). Overall, QRF exerted a clear dose-dependent anti-fibrotic effect at the F3 stage, with high-dose treatment proving more effective than middle- and low-dose regimens. These findings corroborate our previous rat studies (28) and align with current evidence on multi-target natural interventions in CCl_4_-induced fibrosis models (45, 47).

Differential gene expression analysis showed that high-dose QRF significantly corrected aberrant expression of key fibrosis-related genes in the F3-stage model, including *Col1a1, Timp1, Mmp13*, and *Il-18.* These results are consistent with previous proteomic and single-cell transcriptomic studies on HSCs activation (22, 48). High-dose QRF exerted multi-faceted anti-fibrotic effects by inhibiting HSCs activation, reducing ECM deposition, and modulating inflammatory responses. Immunohistochemistry confirmed downregulation of the HSCs marker α-SMA (49), in agreement with reports that Sestrin 2 attenuates HSCs activation *via* the mTOR/AMPK pathway (50). QRF also suppressed Col1a1 protein expression, which correlated with reduced fibrosis (51), and decreased *Timp1* mRNA levels, potentially promoting ECM remodeling, similar to LncRNA-ATB–mediated regulation *via* miR-425-5p (15). Furthermore, high-dose QRF significantly reduced pro-inflammatory cytokines, including IL-1β, IL-18, and TNF-α (52), suggesting that its anti-fibrotic effects involve modulation of inflammatory pathways. Overall, high-dose QRF effectively suppresses HSCs activation, collagen synthesis, and inflammation, thereby exerting robust anti-fibrotic effects, consistent with *in vivo* findings.

This study systematically elucidates the core mechanisms underlying the anti-fibrotic effects of high-dose QRF in F3-stage liver fibrosis. Based on clinical observations suggesting that QRF may regulate the pyroptotic executor protein GSDMD (21), coupled with molecular docking revealing its strong binding affinity to components of the NLRP3 inflammasome, we validated these findings in an animal model. The model confirmed that NLRP3 inflammasome-driven pyroptosis leads to the release of pro-inflammatory cytokines such as IL-1β and IL-18, which exacerbate inflammation and fibrosis (24, 26). Transcriptomic analysis showed that QRF downregulates pyroptosis-related genes and modulates NOD-like receptor signaling pathways, while also reversing macrophage polarization toward the pro-inflammatory M1 phenotype (e.g., decreasing CD80/CD86 expression and increasing Arg1) (53, 54). Subsequent molecular experiments further confirmed that QRF significantly inhibits the mRNA and protein expression of NLRP3, ASC, and Caspase-1. Immunofluorescence studies revealed that macrophages are the primary site of pyroptosis (24, 53), and the NLRP3-driven M1 polarization forms a feed-forward loop that exacerbates pathological damage (55). Importantly, QRF did not alter the total number of macrophages (F4/80), but instead modulated their functional state (56), positioning QRF as an immune modulator. Protein and functional validations further confirmed the occurrence of pyroptosis, as evidenced by the activation of the NLRP3 inflammasome, upregulation of its downstream effectors, p20 Caspase-1 and GSDMD-N, increased TUNEL-positive signals, and elevated levels of IL-1β, IL-18, and LDH release (57). QRF treatment reversed all these indicators and restored the M1/M2 macrophage balance. In conclusion, through a comprehensive research approach involving ‘computational prediction - model development - omics analysis - molecular validation’, this study provides a systematic clarification that QRF targets macrophages, suppresses NLRP3 inflammasome-mediated pyroptosis, and modulates macrophage polarization, thereby improving liver fibrosis.

This study further validated NLRP3-mediated pyroptosis in macrophages at both morphological and functional levels and explored the underlying mechanisms of QRF action. TEM revealed that LPS/ATP stimulation induced hallmark features of pyroptosis (58, 59), including membrane pore formation, cell swelling, membrane rupture, and cytoplasmic leakage. In contrast, treatment with QRF-containing serum preserved membrane integrity and alleviated these ultrastructural abnormalities. These findings are consistent with the *in vivo* results, where QRF suppressed activation of the NLRP3/caspase-1/GSDMD pathway, reduced inflammatory cytokine release, and decreased LDH leakage (60), providing morphological evidence that QRF attenuates pyroptosis. Moreover, these results offer a cellular explanation for the reduced TUNEL-positive signals observed in animal tissues (61). To further confirm the critical role of NLRP3, a functional rescue experiment was performed using the specific agonist Nigericin (NIG). NIG significantly enhanced LPS/ATP-induced NLRP3 inflammasome activation, GSDMD-N formation, and IL-1β/IL-18 release (62), while markedly attenuating the protective effects of QRF on these key markers.

This study elucidates the mechanisms by which QRF acts during F3-stage liver fibrosis. QRF targets liver macrophages, suppressing pyroptosis *via* inhibition of the NLRP3/Caspase-1/GSDMD axis and promoting M1-to-M2 polarization. This reprogramming of the immune microenvironment reduces inflammatory signaling and limits hepatic stellate cell activation. Unlike prior studies examining NLRP3 in hepatocyte (26) or Kupffer cell pyroptosis (53), STING-mediated inflammasome activation (25), and macrophage polarization (55), this work specifically highlights the macrophage NLRP3–pyroptosis axis and its downstream effects on parenchymal injury and stromal activation, advancing current understanding of immune regulation in liver fibrosis. The established F3-stage model provides a useful platform for stage-specific investigation. The proposed ‘QRF–macrophage–NLRP3 pyroptosis axis’ offers a framework for understanding the multi-target actions of TCM and supports strategies targeting the hepatic immune microenvironment. Given the broader anti-inflammatory and anti-fibrotic potential of NLRP3 inhibition (63, 64), these findings may also inform therapeutic approaches for diseases involving NLRP3 activation and macrophage dysfunction.

Based on high-throughput sequencing and our experimental data (**Supplementary Figure S5**), QRF significantly upregulated hepatic miR-223-3p expression. Together with prior dual-luciferase evidence (65), this suggests that miR-223-3p may target and inhibit NLRP3, indicating that QRF regulates macrophage pyroptosis *via* the miR-223-3p/NLRP3 axis to exert anti-fibrotic effects. While this study provides multi-level evidence that QRF suppresses NLRP3 inflammasome–mediated pyroptosis, the upstream regulatory mechanisms remain unclear. Notably, the miR-223-3p/NLRP3 axis was validated only at the tissue level in this study, without functional gain- or loss-of-function studies to establish causality. Future studies should further investigate this mechanism using *in vivo* and *in vitro* models with genetic manipulation approaches.

## 5 Conclusion

**Figure 10:**
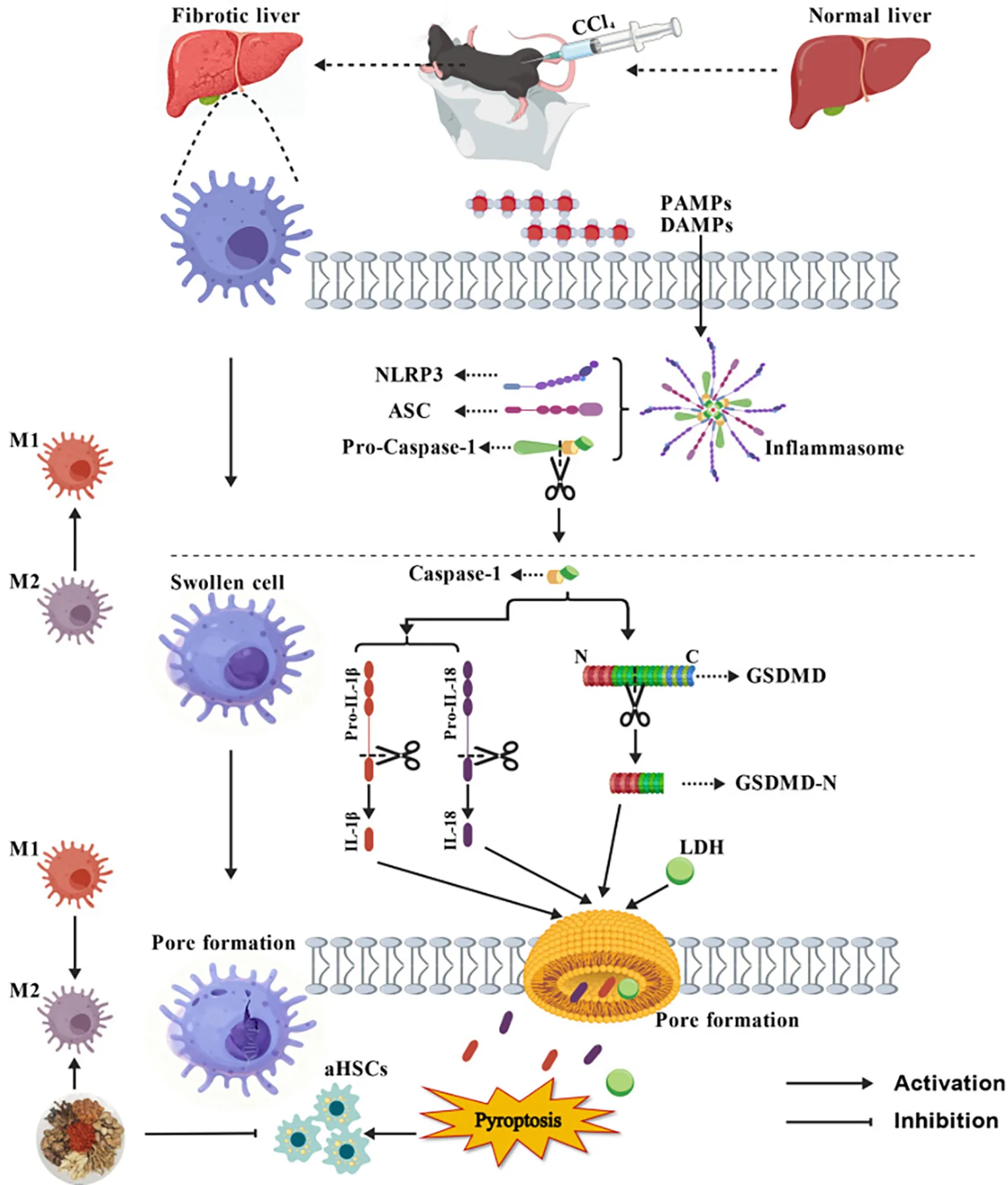
Mechanism of QRF against liver fibrosis: inhibition of the NLRP3/Caspase-1/GSDMD pyroptosis pathway and promotion of M1-to-M2 polarization, leading to attenuated release of IL-1β, IL-18, and LDH.

In summary, QRF exhibits notable antifibrotic efficacy in stage F3 liver fibrosis, primarily through suppression of NLRP3/Caspase-1/GSDMD-mediated macrophage pyroptosis. This effect leads to reduced pro-inflammatory cytokine release and promotes a shift from M1 to M2 macrophage polarization, thereby improving the immune microenvironment and attenuating fibrosis progression (**Figure 10**). Focusing on stage F3, a clinically critical stage for therapeutic intervention, we established a stable and reproducible animal model to validate the efficacy of QRF. By integrating molecular docking, transcriptomic profiling, and both *in vivo* and *in vitro* experiments, we constructed a comprehensive evidence framework identifying macrophage pyroptosis as one of the key mechanisms driving F3 progression. Collectively, these findings delineate key targets and pathways underlying QRF action and provide a mechanistic basis for the development of stage-specific precision therapies.

## Data Availability

The datasets presented in this study can be found in online repositories. The names of the repository/repositories and accession number(s) can be found below: PRJNA1415787 (Bioproject, NCBI).
