# Correction: Qijia Rougan Formula alleviates liver fibrosis by inhibiting NLRP3-mediated pyroptosis and regulating macrophage polarization

**DOI:** 10.3389/fimmu.2026.1945814

**Published:** 2026-09-17

**Authors:** Xin Ding, Baixue Li, Hongfei Song, Chenhao Liu, Zhenglong Zheng, Jibin Liu, Li Wen, Quansheng Feng

**Affiliations:** 1 College of Basic Medical Sciences, Chengdu University of Traditional Chinese Medicine, Chengdu, China; 2 College of Traditional Chinese Medicine, Inner Mongolia Medical University, Hohhot, China

**Keywords:** macrophage polarization, NLRP3 inflammasome, pyroptosis, Qijia Rougan Formula, stage F3 liver fibrosis

Author Peiying Xue was erroneously included as an author in the published article. The correct author list reads:

“Xin Ding^1,2^, Baixue Li^1^, Hongfei Song^1^, Chenhao Liu^1^, Zhenglong Zheng^1^, Jibin Liu^1*^, Li Wen^1*^ and Quansheng Feng^1*^”

“1. College of Basic Medical Sciences, Chengdu University of Traditional Chinese Medicine, Chengdu, China

2. College of Traditional Chinese Medicine, Inner Mongolia Medical University, Hohhot, China”

In addition, author Peiying Xue was erroneously assigned as equal contributing author.

The **Author contributions** Statement has been corrected to read:

“XD: Conceptualization, Data curation, Formal analysis, Funding acquisition, Methodology, Resources, Writing – original draft. BL: Project administration, Writing – original draft. HS: Investigation, Writing – original draft. CL: Formal analysis, Software, Writing – original draft. ZZ: Data curation, Formal analysis, Writing – original draft. JL: Conceptualization, Funding acquisition, Supervision, Writing – review & editing. LW: Methodology, Supervision, Writing – review & editing. QF: Funding acquisition, Project administration, Supervision, Writing – review & editing.”

There was a mistake in **Figure 7E** as published. The molecular weight annotations of the Western blot bands were incorrectly labeled in the original version. The corrected **Figure 7** appears below.

**Figure 7:**
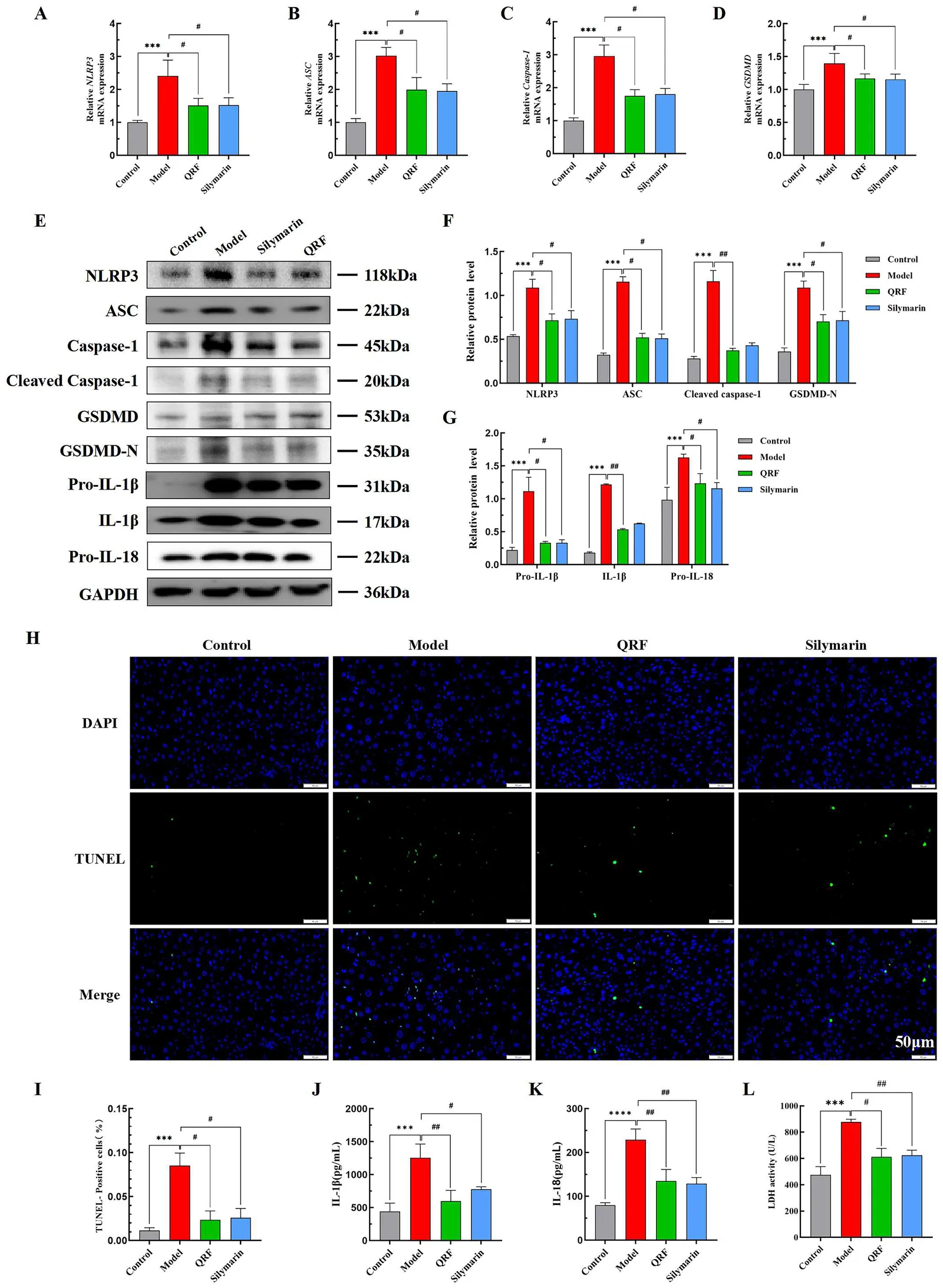
QRF alleviates inflammation, pyroptosis, and fibrosisin stage F3 liver fibrosis. **(A–D)** qRT–PCR analysis of *NLRP3* inflammasome-related gene: *Nlrp3*, *Caspase-1, ASC*, and *GSDMD* (normalized to *Gapdh*). **(E)** Representative Western blots ofNLRP3, Pro-Caspase-1, cleaved Caspase-1 (p20), ASC, GSDMD, GSDMD-N, Pro-IL-1β, mature IL-1β, and Pro-IL-18. GAPDH asloading control. **(F, G)** Densitometric analysis of cleaved Caspase-1(p20), ASC, GSDMD-N, Pro-IL-1β, mature IL-1β, and Pro-IL-18. **(H)** TUNEL staining (green) in liver sections; nuclei counterstainedwith DAPI (blue). (20bl scale bar: 50 μm). **(I)** Quantification ofTUNEL-positive area. **(J, K)** Hepatic IL-1β and IL-18 levels measured by ELISA. **(L)** LDH levels in supernatants. Data are presentedas mean ± SD (n = 3–6 mice per group). ***p < 0.001, ****p < 0.0001 vs. Control; ^#^p < 0.05, ^##^p < 0.01 vs. Model.

**Figure 7** | QRF alleviates inflammation, pyroptosis, and fibrosis in stage F3 liver fibrosis. (A–D) qRT–PCR analysis of NLRP3 inflammasome-related gene: Nlrp3, Caspase-1, ASC, and GSDMD (normalized to Gapdh). (E) Representative Western blots of NLRP3, Pro-Caspase-1, cleaved Caspase-1 (p20), ASC, GSDMD, GSDMD-N, Pro-IL-1β, mature IL-1β, and Pro-IL-18. GAPDH as loading control. (F, G) Densitometric analysis of cleaved Caspase-1 (p20), ASC, GSDMD-N, Pro-IL-1β, mature IL-1β, and Pro-IL-18. (H) TUNEL staining (green) in liver sections; nuclei counterstained with DAPI (blue). (20bl scale bar: 50 µm). (I) Quantification of TUNEL-positive area. (J, K) Hepatic IL-1β and IL-18 levels measured by ELISA. (L) LDH levels in supernatants. Data are presented as mean ± SD (n = 3–6 mice per group). ***p < 0.001, ****p < 0.0001 vs. Control; #p < 0.05, ##p < 0.01 vs. Model.

There was a mistake in **Figure 9C** as published. The group labels in the qPCR results were inadvertently duplicated in the original version. The corrected **Figure 9** appears below.

**Figure 9:**
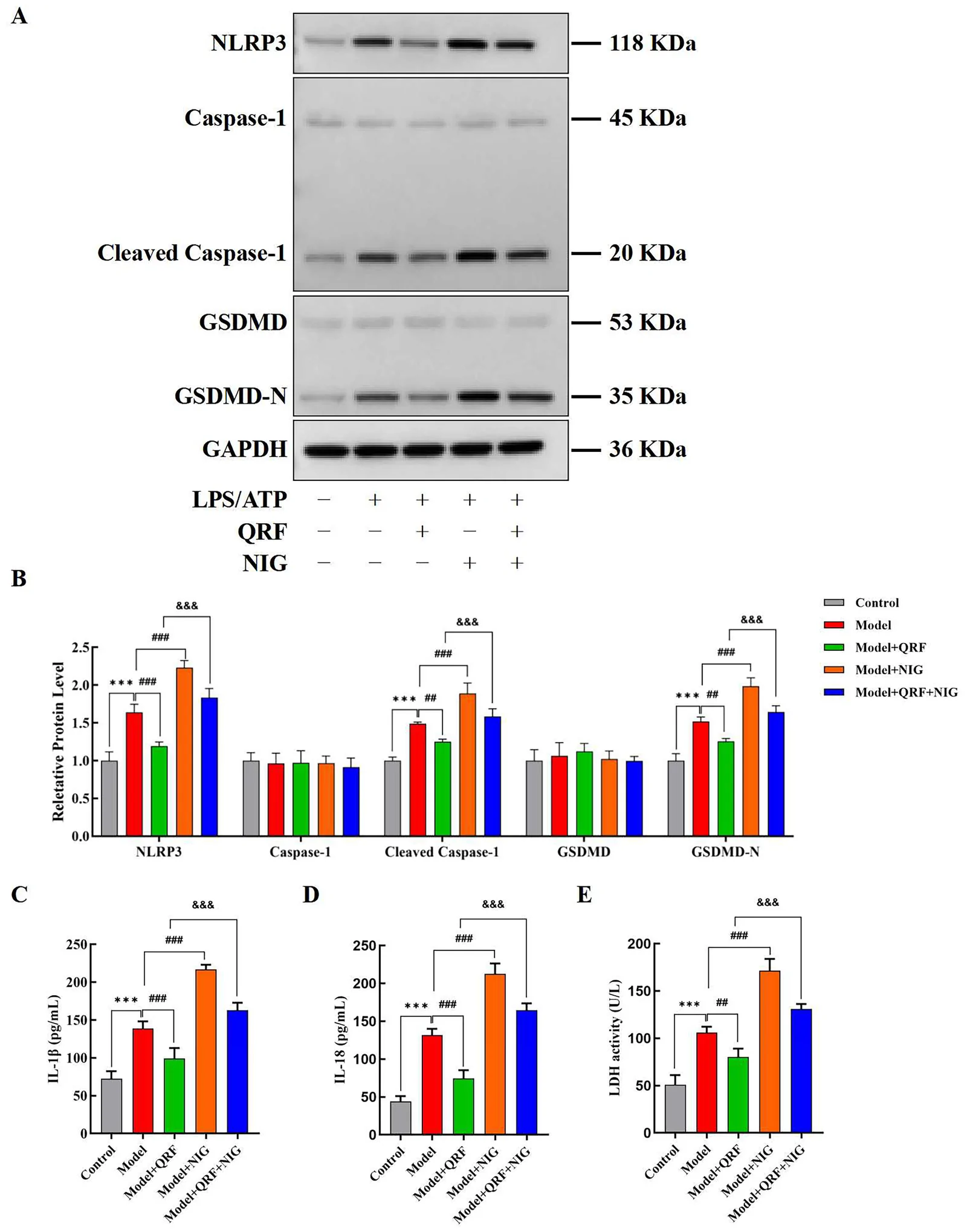
NLRP3 agonist NIG attenuates the inhibitory effect of QRF on macrophage pyroptosis. **(A)** Representative Western blot images of NLRP3, Pro-Caspase-1, cleaved Caspase-1 (p20), full-length GSDMD, and GSDMD-N protein expression in each group. GAPDH served as the loading control. **(B)** Densitometric analysis of band intensity for NLRP3, Pro-Caspase-1, cleaved Caspase-1 (p20), full-length GSDMD, and GSDMD-N. **(C–E)** Concentrations of **(C)** IL-1β and **(D)** IL-18 in cell supernatant, and **(E)** LDH release rate. Data are presented as mean ± SD (n = 3). ***p < 0.001 vs. Control; ^##^p < 0.01, ^###^p < 0.001 vs. Model; ^&&&^p < 0.001 vs. Model + QRF serum group.

**Figure 9** | NLRP3 agonist NIG attenuates the inhibitory effect of QRF on macrophage pyroptosis. (A) Representative Western blot images of NLRP3, Pro-Caspase-1, cleaved Caspase-1 (p20), full-length GSDMD, and GSDMD-N protein expression in each group. GAPDH served as the loading control. (B) Densitometric analysis of band intensity for NLRP3, Pro-Caspase-1, cleaved Caspase-1 (p20), full-length GSDMD, and GSDMD-N. (C–E) Concentrations of (C) IL-1β and (D) IL-18 in cell supernatant, and (E) LDH release rate. Data are presented as mean ± SD (n = 3). ***p < 0.001 vs, Control; ##p < 0.01, ###p < 0.001 vs, Model; &&&p < 0.001 vs, Model + QRF serum group.

The original version of this article has been updated.

