# Supplementary material for: Qijia Rougan Formula alleviates liver fibrosis by inhibiting NLRP3-mediated pyroptosis and regulating macrophage polarization
Source: Front Immunol. 2026 May 5;17:1796042. doi: 10.3389/fimmu.2026.1796042 (PMC13183564; doi:10.3389/fimmu.2026.1796042)
Supplement: Supplementary file 1 [file DataSheet1.docx]

**Qijia Rougan Formula alleviates liver fibrosis by inhibiting NLRP3-mediated pyroptosis and regulating macrophage polarization**
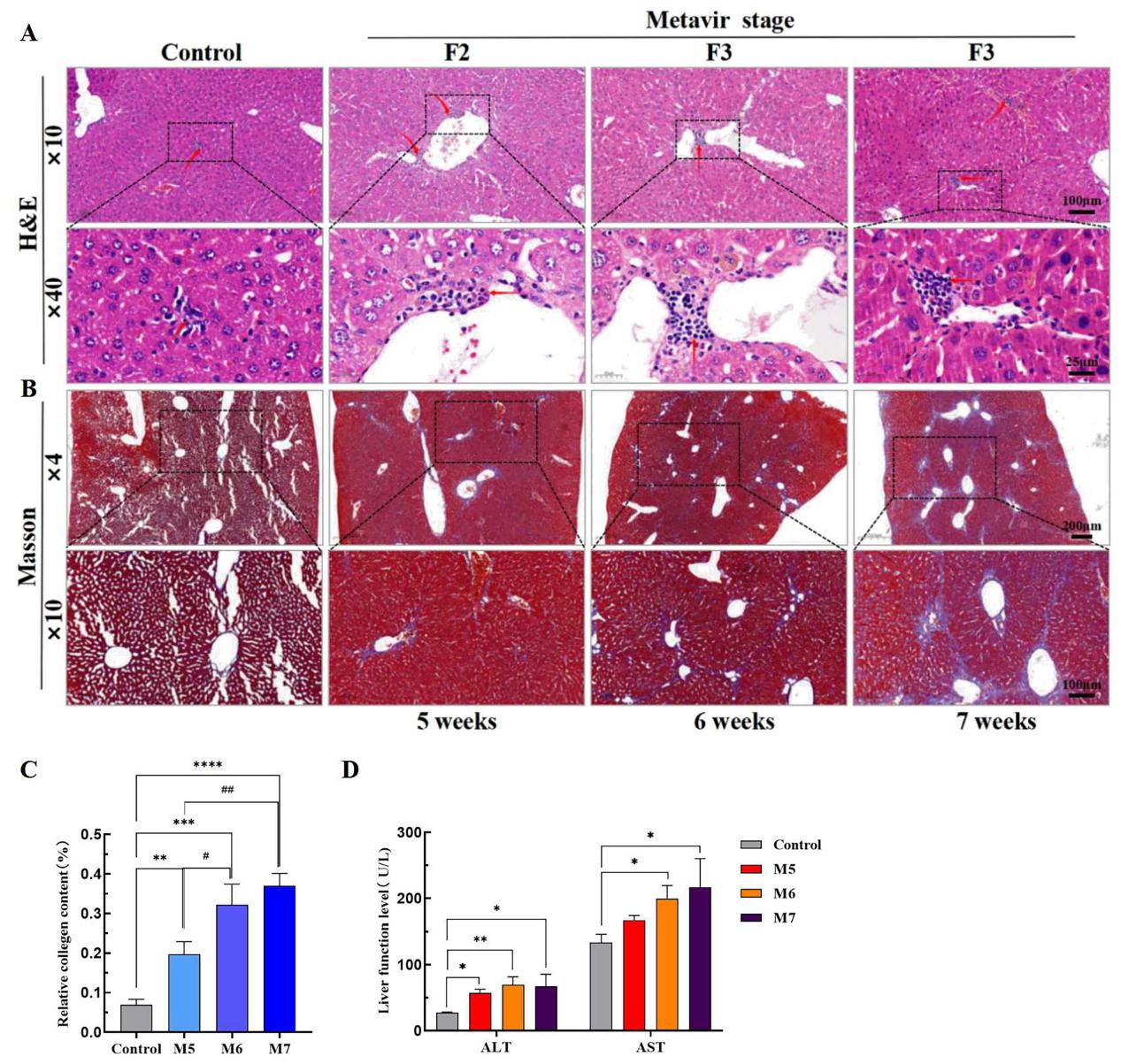


**FIGURE S1**

Liver injury and fibrosis progressively worsened from weeks 5 to 7 post-modeling. **(A)** Representative hematoxylin and eosin (H&E) staining of liver tissues (scale bars: 100 μm, ×10; 25 μm, ×40). **(B)** Representative Masson’s trichrome staining (scale bars: 200 μm, ×4; 100 μm, ×10). **(C)** Quantitative analysis of liver fibrosis area. **(D)** Serum alanine aminotransferase (ALT) and aspartate aminotransferase (AST) levels. Data are expressed as mean ± SD (n = 3 mice per group). *p < 0.05, **p < 0.01, ***p < 0.001, ****p < 0.0001 compared to Control; ^#^p < 0.05, ^##^p < 0.01 compared to week 5 group. Abbreviations: F, fibrosis area; M, model group.

**
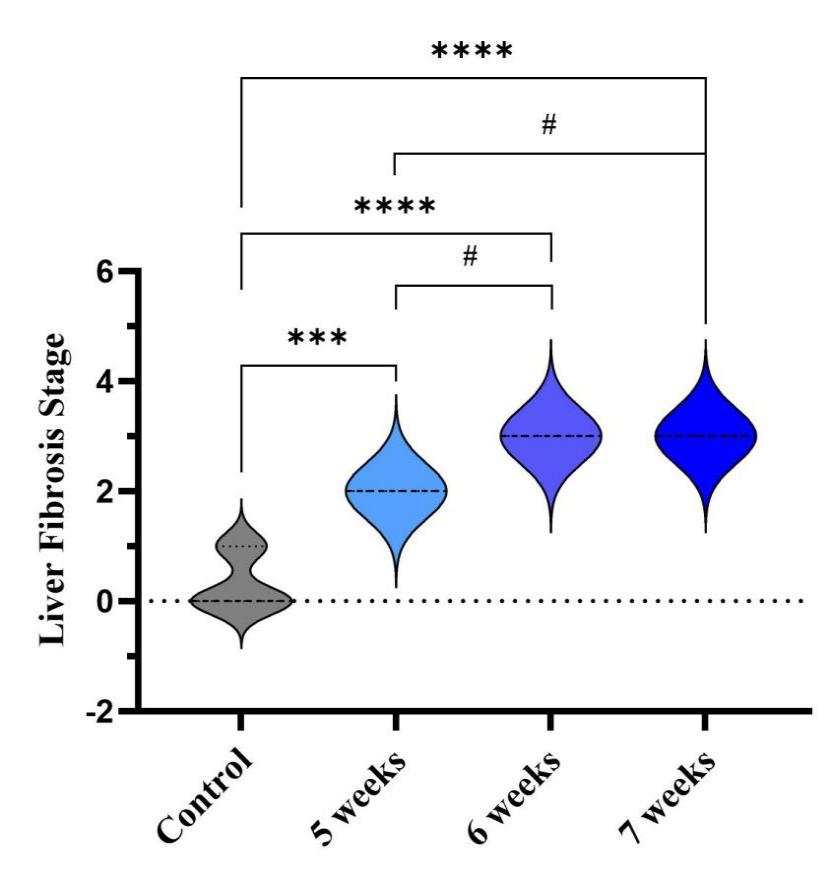
**

**FIGURE S2**

Progression of liver fibrosis stages in the mouse model from weeks 5 to 7. Data are presented as mean ± SD (n = 3 mice per group). ***p < 0.001, ****p < 0.0001 compared to Control; ^#^p < 0.05 compared to week 5 group

**TABLE S1**

**Histopathological staging and scoring of liver fibrosis in mice at weeks 5, 6, and 7.**

| **Grading of Mouse Liver Fibrosis** | | | | | | | **Degree of inflammation** | | | | |
| --- | --- | --- | --- | --- | --- | --- | --- | --- | --- | --- | --- |
| **Group** | **SSS Scoring System for Liver Fibrosis** | | | | | **Metavir Systems Liver Fibrosis Staging** | **Semi-Quantitative Inflammatory**  **Activity Scoring System** | | | | |
|  | **L** | **P** | **N** | **W** | **Scoring** |  | **P** | **L** | **PN** | **BN** | **Scoring** |
| **Ctr-1** | 0 | 0 | 0 | 0 | 0 | 0 | 0 | 0 | 0 | 0 | 0 |
| **Ctr-2** | 0 | 0 | 0 | 0 | 0 | 0 | 1 | 0 | 0 | 0 | 1 |
| **Ctr-3** | 1 | 1 | 0 | 0 | 2 | 1 | 1 | 0 | 0 | 0 | 1 |
| **M-WK5-1** | 2 | 2 | 1 | 1 | 6 | 2 | 1 | 1 | 0 | 0 | 2 |
| **M-WK5-2** | 2 | 2 | 1 | 2 | 8 | 2 | 3 | 1 | 0 | 0 | 4 |
| **M-WK5-3** | 2 | 2 | 1 | 1 | 6 | 2 | 3 | 1 | 0 | 0 | 4 |
| **M-WK6-1** | 2 | 2 | 2 | 2 | 12 | 3 | 3 | 3 | 0 | 0 | 6 |
| **M-WK6-2** | 2 | 2 | 2 | 2 | 12 | 3 | 3 | 1 | 0 | 0 | 4 |
| **M-WK6-3** | 2 | 2 | 2 | 2 | 12 | 3 | 1 | 1 | 0 | 0 | 2 |
| **M-WK7-1** | 2 | 2 | 2 | 2 | 12 | 3 | 3 | 3 | 1 | 1 | 10 |
| **M-WK7-2** | 2 | 2 | 2 | 2 | 12 | 3 | 3 | 1 | 0 | 0 | 4 |
| **M-WK7-3** | 2 | 2 | 2 | 2 | 12 | 3 | 1 | 1 | 0 | 0 | 2 |

Abbreviations: Ctr, Control; M, Model; WK, Week; L, Lobular; P, Portal area; N, Number; W, Width; PN, Piecemeal Necrosis；BN, Bridging Necrosis.

**
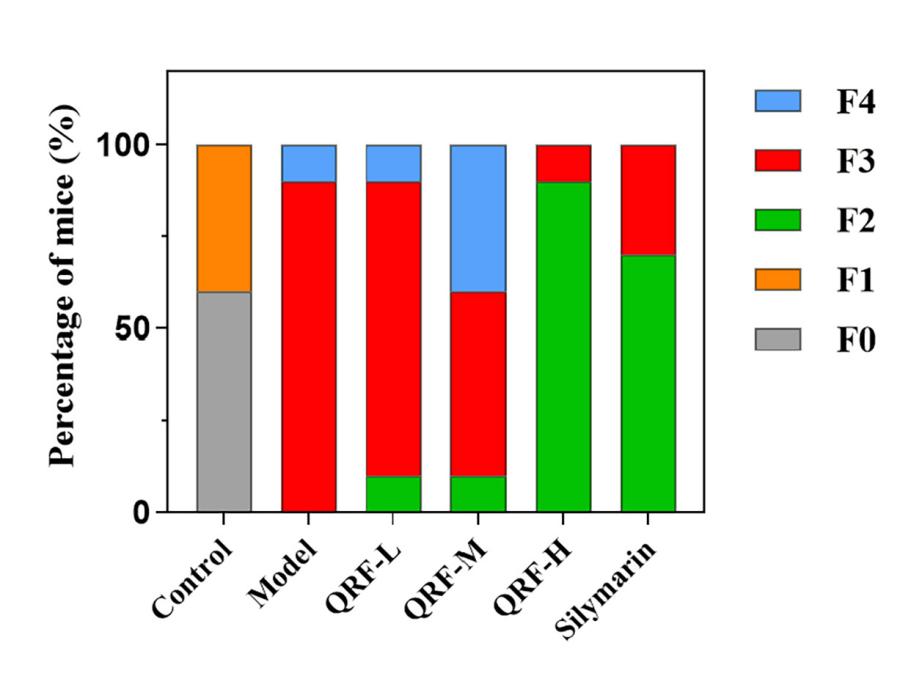
**

**FIGURE S3**

Distribution of Hepatic Fibrosis Stages Among Different Treatment Groups. Mice were subjected to a 6-week fibrosis induction period followed by a 6-week treatment regimen. Liver fibrosis was staged histologically from F0 (no fibrosis) to F4 (severe cirrhosis). Data are presented as the percentage of mice within each group (n=10 mice per group) at each fibrosis stage. Control: vehicle control; Model: CCl₄-induced model; QRF-L/M/H: low, medium, and high dose of QRF treatment; Silymarin: positive control drug.

**
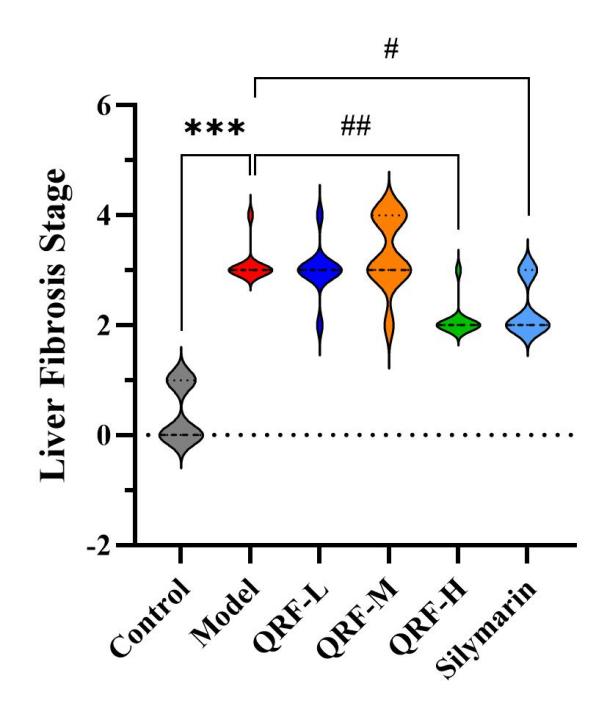
**

**FIGURE S4**

Comparison of Hepatic Fibrosis Stage Scores Across Groups. Mice were subjected to a 6-week fibrosis induction period followed by a 6-week treatment regimen. Liver fibrosis was staged histologically from F0 (no fibrosis) to F4 (severe cirrhosis). Data are presented as mean ± SD (n=10 mice per group). ***p < 0.001 compared to Control; #p < 0.05, ##p < 0.01 compared to Model. Control: vehicle control; Model: CCl₄-induced model; QRF-L/M/H: low, medium, and high dose of QRF treatment; Silymarin: positive control drug.

**TABLE S2**

**Pathological assessment of F3-stage fibrotic liver after treatment with low-, medium-, and high-dose QRF.**

| **Grading of Mouse Liver Fibrosis** | | | | | | | **Degree of inflammation** | | | | |
| --- | --- | --- | --- | --- | --- | --- | --- | --- | --- | --- | --- |
| **Group** | **SSS Scoring System for Liver Fibrosis** | | | | | **Metavir Systems Liver Fibrosis Staging** | **Semi-Quantitative Inflammatory**  **Activity Scoring System** | | | | |
|  | **L** | **P** | **N** | **W** | **Scoring** |  | **P** | **L** | **PN** | **BN** | **Scoring** |
| **Ctr-1** | 1 | 1 | 0 | 0 | 2 | 1 | 1 | 1 | 0 | 0 | 2 |
| **Ctr-2** | 0 | 0 | 0 | 0 | 0 | 0 | 0 | 0 | 0 | 0 | 0 |
| **Ctr-3** | 0 | 0 | 0 | 0 | 0 | 0 | 1 | 0 | 0 | 0 | 1 |
| **Ctr-4** | 1 | 1 | 0 | 0 | 2 | 1 | 1 | 0 | 0 | 0 | 1 |
| **Ctr-5** | 2 | 1 | 0 | 0 | 3 | 1 | 0 | 1 | 0 | 0 | 1 |
| **Ctr-6** | 0 | 0 | 0 | 0 | 0 | 0 | 3 | 1 | 0 | 0 | 4 |
| **Ctr-7** | 0 | 0 | 0 | 0 | 0 | 0 | 1 | 1 | 0 | 0 | 2 |
| **Ctr-8** | 0 | 0 | 0 | 0 | 0 | 0 | 3 | 1 | 0 | 0 | 4 |
| **Ctr-9** | 0 | 0 | 0 | 0 | 0 | 0 | 0 | 1 | 0 | 0 | 1 |
| **Ctr-10** | 0 | 1 | 0 | 0 | 1 | 1 | 1 | 1 | 0 | 0 | 2 |
| **M-1** | 2 | 2 | 2 | 2 | 12 | 3 | 2 | 2 | 2 | 2 | 12 |
| **M-2** | 2 | 2 | 2 | 2 | 12 | 3 | 2 | 2 | 2 | 2 | 12 |
| **M-3** | 2 | 2 | 2 | 4 | 20 | 3 | 2 | 2 | 2 | 4 | 20 |
| **M-4** | 2 | 2 | 2 | 2 | 12 | 3 | 2 | 2 | 2 | 2 | 12 |
| **M-5** | 2 | 2 | 2 | 2 | 12 | 3 | 2 | 2 | 2 | 2 | 12 |
| **M-6** | 2 | 2 | 2 | 4 | 20 | 3 | 2 | 2 | 2 | 4 | 20 |
| **M-7** | 2 | 2 | 2 | 2 | 12 | 3 | 3 | 3 | 1 | 1 | 10 |
| **M-8** | 2 | 2 | 2 | 3 | 16 | 3 | 3 | 3 | 3 | 1 | 14 |
| **M-9** | 2 | 3 | 3 | 4 | 29 | 4 | 3 | 3 | 3 | 3 | 18 |
| **M-10** | 2 | 2 | 2 | 2 | 12 | 3 | 3 | 3 | 3 | 1 | 14 |
| **QRF-L-1** | 2 | 2 | 2 | 2 | 12 | 3 | 3 | 3 | 0 | 0 | 6 |
| **QRF-L-2** | 2 | 2 | 2 | 3 | 16 | 3 | 3 | 3 | 3 | 1 | 14 |
| **QRF-L-3** | 2 | 2 | 2 | 3 | 16 | 3 | 3 | 3 | 3 | 1 | 14 |
| **QRF-L-4** | 2 | 2 | 2 | 2 | 12 | 3 | 3 | 3 | 3 | 1 | 14 |
| **QRF-L-5** | 2 | 2 | 2 | 1 | 8 | 3 | 3 | 3 | 3 | 1 | 14 |
| **QRF-L-6** | 2 | 2 | 1 | 1 | 6 | 2 | 3 | 3 | 1 | 0 | 8 |
| **QRF-L-7** | 2 | 2 | 2 | 2 | 12 | 3 | 3 | 3 | 1 | 1 | 10 |
| **QRF-L-8** | 2 | 2 | 2 | 2 | 12 | 3 | 3 | 3 | 3 | 1 | 14 |
| **QRF-L-9** | 2 | 2 | 2 | 2 | 12 | 3 | 3 | 3 | 1 | 0 | 8 |
| **QRF-L-10** | 2 | 3 | 3 | 4 | 29 | 4 | 3 | 3 | 3 | 1 | 14 |
| **QRF-M-1** | 2 | 2 | 2 | 3 | 16 | 3 | 3 | 3 | 3 | 1 | 14 |
| **QRF-M-2** | 2 | 2 | 2 | 3 | 16 | 3 | 3 | 3 | 3 | 1 | 14 |
| **QRF-M-3** | 2 | 3 | 3 | 4 | 29 | 4 | 3 | 3 | 3 | 3 | 18 |
| **QRF-M-4** | 2 | 2 | 2 | 2 | 12 | 3 | 3 | 3 | 1 | 3 | 14 |
| **QRF-M-5** | 2 | 2 | 2 | 4 | 20 | 3 | 3 | 3 | 1 | 1 | 10 |
| **QRF-M-6** | 2 | 3 | 3 | 4 | 29 | 4 | 3 | 3 | 3 | 3 | 18 |
| **QRF-M-7** | 2 | 2 | 2 | 3 | 16 | 3 | 3 | 3 | 1 | 1 | 10 |
| **QRF-M-8** | 2 | 3 | 3 | 4 | 29 | 4 | 3 | 4 | 3 | 3 | 19 |
| **QRF-M-9** | 2 | 3 | 3 | 4 | 29 | 4 | 3 | 3 | 3 | 1 | 14 |
| **QRF-M-10** | 2 | 2 | 1 | 1 | 6 | 2 | 3 | 3 | 1 | 1 | 10 |
| **QRF-H-1** | 2 | 2 | 1 | 1 | 6 | 2 | 3 | 1 | 0 | 0 | 4 |
| **QRF-H-2** | 2 | 2 | 1 | 1 | 6 | 2 | 3 | 3 | 0 | 0 | 6 |
| **QRF-H-3** | 2 | 2 | 1 | 1 | 6 | 2 | 3 | 3 | 1 | 1 | 10 |
| **QRF-H-4** | 2 | 2 | 1 | 1 | 6 | 2 | 3 | 3 | 1 | 0 | 8 |
| **QRF-H-5** | 2 | 2 | 1 | 1 | 6 | 2 | 3 | 3 | 0 | 0 | 6 |
| **QRF-H-6** | 2 | 2 | 2 | 2 | 12 | 3 | 3 | 3 | 0 | 0 | 6 |
| **QRF-H-7** | 2 | 2 | 1 | 1 | 6 | 2 | 3 | 3 | 0 | 0 | 6 |
| **QRF-H-8** | 2 | 2 | 1 | 1 | 6 | 2 | 3 | 3 | 0 | 0 | 6 |
| **QRF-H-9** | 2 | 2 | 1 | 1 | 6 | 2 | 3 | 3 | 0 | 0 | 6 |
| **QRF-H-10** | 2 | 2 | 1 | 1 | 6 | 2 | 3 | 1 | 0 | 0 | 4 |
| **Silymarin-1** | 2 | 2 | 1 | 1 | 6 | 2 | 3 | 1 | 0 | 0 | 4 |
| **Silymarin-2** | 2 | 2 | 2 | 2 | 12 | 3 | 3 | 3 | 1 | 0 | 8 |
| **Silymarin-3** | 2 | 2 | 1 | 1 | 6 | 2 | 3 | 3 | 3 | 1 | 14 |
| **Silymarin-4** | 2 | 2 | 1 | 1 | 6 | 2 | 3 | 1 | 0 | 0 | 4 |
| **Silymarin-5** | 2 | 2 | 1 | 2 | 8 | 2 | 3 | 3 | 0 | 0 | 6 |
| **Silymarin-6** | 2 | 2 | 2 | 2 | 12 | 3 | 3 | 3 | 0 | 0 | 6 |
| **Silymarin-7** | 2 | 2 | 1 | 1 | 6 | 2 | 3 | 3 | 0 | 0 | 6 |
| **Silymarin-8** | 2 | 2 | 2 | 1 | 8 | 3 | 3 | 3 | 0 | 0 | 6 |
| **Silymarin-9** | 2 | 2 | 1 | 1 | 6 | 2 | 3 | 3 | 1 | 0 | 8 |
| **Silymarin-10** | 2 | 2 | 1 | 1 | 6 | 2 | 1 | 3 | 1 | 0 | 6 |

Abbreviations: F, Fibrosis; Ctr, Control; M, Model; QRF-L, Qijia Rougan Formula Low dose；QRF-M, Qijia Rougan Formula Medium dose；QRF-H, Qijia Rougan Formula High dose; WK, Week; L, Lobular; P, Portal area; N, Number; W, Width; PN, Piecemeal Necrosis; BN, Bridging Necrosis.


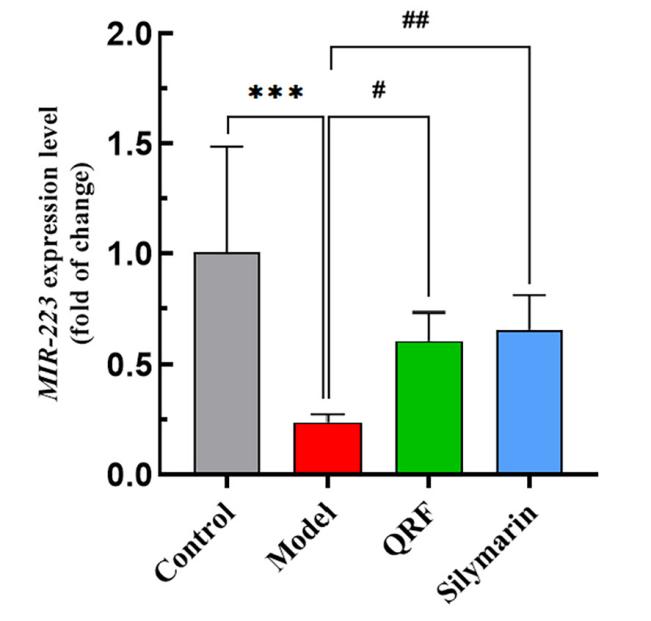


**FIGURE S5**

Relative expression level of miR-223 in liver tissues of mice from different groups.The expression level of miR-223 in the liver was determined by qRT-PCR in the Control, Model, QRF-treated, and Silymarin-treated (positive control) groups. Data are presented as mean ± SD (n = 6 per group). ***p < 0.001 compared to Control; ^#^p < 0.05, ^##^p < 0.01 compared to Model.
